# Mint3-depletion-induced energy stress sensitizes triple-negative breast cancer to chemotherapy via HSF1 inactivation

**DOI:** 10.1038/s41419-023-06352-4

**Published:** 2023-12-11

**Authors:** Noritaka Tanaka, Hikari Okada, Kiyoshi Yamaguchi, Masahide Seki, Daisuke Matsubara, Noriko Gotoh, Yutaka Suzuki, Yoichi Furukawa, Taro Yamashita, Jun-ichiro Inoue, Shuichi Kaneko, Takeharu Sakamoto

**Affiliations:** 1 Department of Cancer Biology, Institute of Biomedical Science, Kansai Medical University, Osaka, Japan; 2 Information-Based Medicine Development, Graduate School of Medical Sciences, Kanazawa University, Ishikawa, Japan; 3 Division of Clinical Genome Research, the Institute of Medical Science, The University of Tokyo, Tokyo, Japan; 4 Department of Computational Biology and Medical Sciences, Graduate School of Frontier Sciences, The University of Tokyo, Chiba, Japan; 5 Department of Pathology, University of Tsukuba, Ibaraki, Japan; 6 Division of Cancer Cell Biology, Cancer Research Institute, Kanazawa University, Ishikawa, Japan; 7 Department of System Biology, Institute of Medical, Pharmaceutical and Health Sciences, Kanazawa University, Ishikawa, Japan; 8 The University of Tokyo Pandemic Preparedness, Infection and Advanced Research Center (UTOPIA), Tokyo, Japan

**Keywords:** Breast cancer, Chemotherapy

## Abstract

Given the lack of therapeutic targets, the conventional approach for managing triple-negative breast cancer (TNBC) involves the utilization of cytotoxic chemotherapeutic agents. However, most TNBCs acquire resistance to chemotherapy, thereby lowering the therapeutic outcome. In addition to oncogenic mutations in TNBC, microenvironment-induced mechanisms render chemoresistance more complex and robust in vivo. Here, we aimed to analyze whether depletion of Munc18-1 interacting protein 3 (Mint3), which activates hypoxia-inducible factor 1 (HIF-1) during normoxia, sensitizes TNBC to chemotherapy. We found that Mint3 promotes the chemoresistance of TNBC in vivo. Mint3 depletion did not affect the sensitivity of human TNBC cell lines to doxorubicin and paclitaxel in vitro but sensitized tumors of these cells to chemotherapy in vivo. Transcriptome analyses revealed that the Mint3–HIF-1 axis enhanced heat shock protein 70 (HSP70) expression in tumors of TNBC cells. Administering an HSP70 inhibitor enhanced the antitumor activity of doxorubicin in TNBC tumors, similar to Mint3 depletion. Mint3 expression was also correlated with HSP70 expression in human TNBC specimens. Mechanistically, Mint3 depletion induces glycolytic maladaptation to the tumor microenvironment in TNBC tumors, resulting in energy stress. This energy stress by Mint3 depletion inactivated heat shock factor 1 (HSF-1), the master regulator of HSP expression, via the AMP-activated protein kinase/mechanistic target of the rapamycin pathway following attenuated HSP70 expression. In conclusion, Mint3 is a unique regulator of TNBC chemoresistance in vivo via metabolic adaptation to the tumor microenvironment, and a combination of Mint3 inhibition and chemotherapy may be a good strategy for TNBC treatment.

## Introduction

In 2020, breast cancer, one of the most prevalent malignancies in women, resulted in 2.3 million new diagnoses and 685,000 fatalities worldwide. Breast cancers are classified into four subtypes depending on the expression levels of human epidermal growth factor receptor-2 and hormone receptors, including estrogen receptors and progesterone receptors: luminal A, luminal B, epidermal growth factor receptor-2 positive, and triple-negative subtypes [1]. Triple-negative breast cancer (TNBC) is a subtype of breast cancer that lacks estrogen and progesterone receptors and epidermal growth factor receptor-2 and comprises ~15–20% of all breast cancers. Due to the lack of therapeutic targets, chemotherapy with cytotoxic drugs is usually used for TNBC treatment. However, most TNBCs acquire resistance to chemotherapy, and the therapeutic outcome of TNBC after chemotherapy remains low.

Oncogenic mutations confer chemoresistance to cancer cells in various ways. Oncogene-induced or genetically amplified ATP-binding cassette transporters eject cytotoxic drugs from cancer cells, resulting in multidrug resistance [2]. Cell survival signals and increased expression of anti-apoptotic molecules increase the survival of cancer cells after chemotherapy [3]. Heat shock factor (HSF) and its target heat shock proteins (HSPs) also maintain proteostasis against cytotoxic reagents, thereby increasing the survival of cancer cells [4–6]. These chemoresistance traits in cancer cells are well-reconstituted in vitro. However, the sensitivity to chemotherapy in vitro does not always reflect that in vivo. Microenvironment-induced mechanisms, such as epigenetic regulation adapting to the tumor microenvironment, tumor-stroma interaction, and physical barriers of the extracellular matrix, make chemoresistance more complex and robust in vivo [7]. Hypoxia is a major cause of microenvironment-dependent chemoresistance. Under hypoxic conditions, hypoxia-inducible factor 1 (HIF-1), an essential transcription factor for hypoxic responses, is activated and promotes the adaptation of its target genes to hypoxic conditions [8–10]. Among the HIF-1 target genes, glycolysis-related genes, such as *SLC2A1*, *HK2*, *PKM*, and *LDHA* contribute to switching the energy production machinery in cells from mitochondrial respiration to glycolysis. Glycolytic enzymes and metabolites have been reported to promote chemoresistance in various cancer types [11–14]. Interestingly, cancer cells upregulate glycolysis even during normoxia. This phenomenon is known as the Warburg effect [15–18]. HIF-1 is usually suppressed under normoxic conditions, but oncogenic signaling pathways, such as the Ras signaling pathway and the PI3K/AKT signaling pathway, enhance HIF-1 expression even during normoxia [8–10].

In addition to oncogenic signaling pathways, Munc18-1-interacting protein 3 (Mint3; also known as amyloid precursor protein-binding family A member 3) activates the transcriptional activity of HIF-1 during normoxia in cancer cells [19–24]. Mint3 suppresses oxygen-dependent HIF-1 inhibitory hydroxylase, factor-inhibiting HIF-1 (FIH-1), by binding to it. Mint3 depletion attenuates the Warburg effect in various cancer cells, including the TNBC cell line MDA-MB-231, in vitro [21]. Mint3 depletion also attenuates tumor growth and reduces angiogenesis in mouse xenograft models [19–21, 24, 25]. Furthermore, Mint3 depletion attenuated the chemoresistance of pancreatic cancer cells both in vitro and in vivo [19]. This mechanism depends on S-phase kinase-associated protein 2 expression via the Mint3-HIF-1 axis and is specific to pancreatic cancer. Mint3 depletion did not affect S-phase kinase-associated protein 2 expression in the human TNBC cell line MDA-MB-231. Interestingly, the administration of naphthofluorescein, which inhibits Mint3-mediated HIF-1 activation, enhanced the antitumor effects of gemcitabine and doxorubicin in pancreatic cancer and TNBC tumors, respectively, in mouse xenograft models [26]. These results indicate that Mint3 may also contribute to the chemoresistance of TNBC, at least in vivo. However, the mechanism by which Mint3 promotes chemoresistance in TNBC remains unclear. To address this, we analyzed whether Mint3 depletion sensitizes TNBC to chemotherapy in both in vitro and in vivo experiments.

## Results

### Mint3 depletion does not affect chemoresistance in vitro in TNBC cell lines

**Fig. 1:**
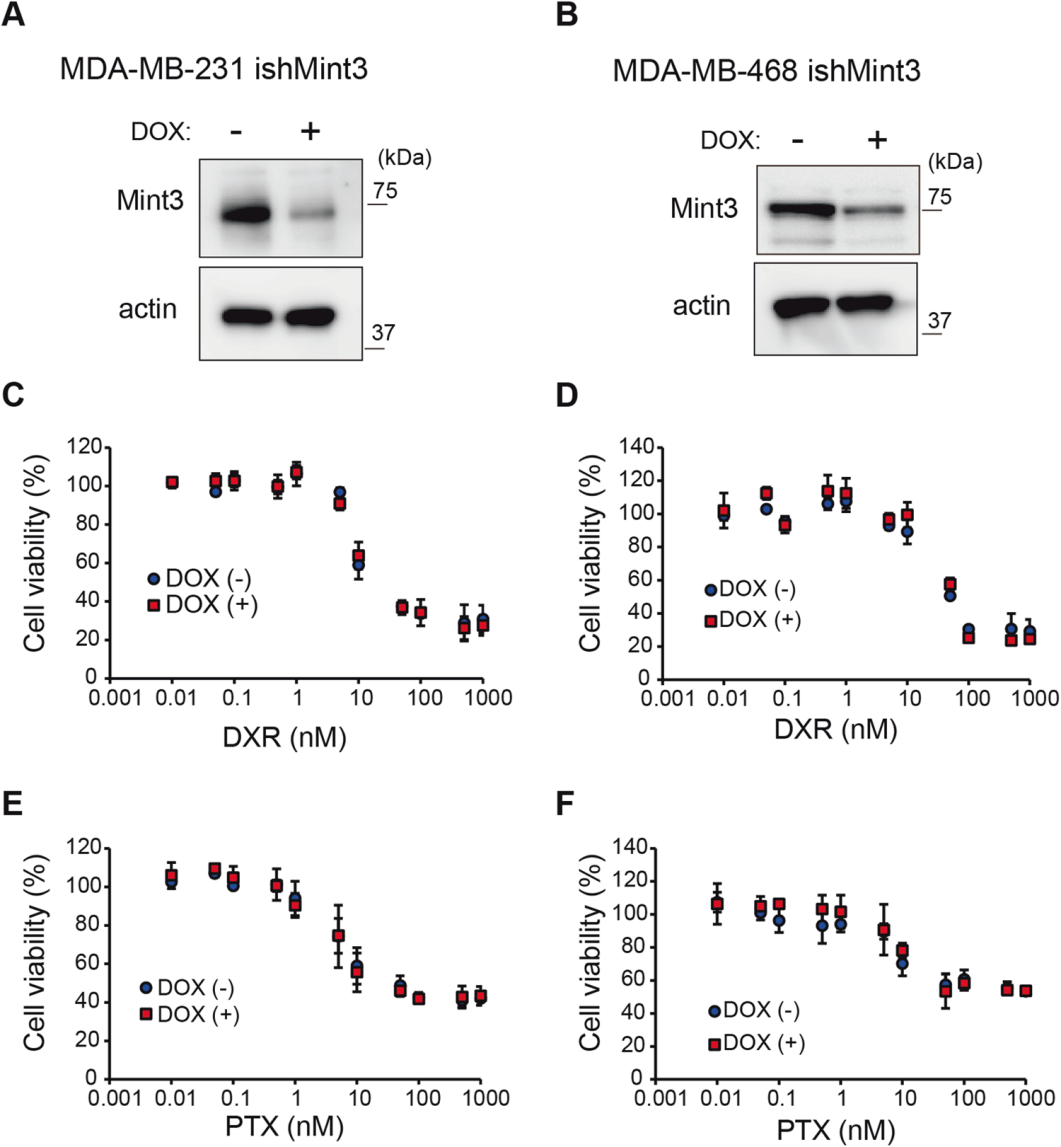
Mint3 depletion does not affect chemoresistance in vitro in TNBC cell lines. **A**, **B** Expression of Mint3 in MDA-MB-231 (**A**) and MDA-MB-468 cells (**B**) expressing doxycycline-inducible shRNAs against Mint3 (ishMint3) treated with doxycycline (1 μg/ml) for 3 days. **C**, **D** Cytotoxicity assay of control (DOX(−)) and doxycycline-pre-treated (DOX (+)) MDA-MB-231 (**C**) and MDA-MB-468 (**D**) ishMint3 cells in the presence of doxorubicin (DXR) for 24 h at the indicated concentration. **E**, **F** Cytotoxicity assay of control (DOX(−)) and doxycycline-pre-treated (DOX(+)) MDA-MB-231 (**E**) and MDA-MB-468 (**F**) ishMint3 cells in the presence of paclitaxel (PTX) for 24 h at the indicated concentration. In **C**–**F**, the data represent the mean ± SD (*n* = 3).

Based on the molecular classification of breast cancer, TNBC encompasses the basal and claudin-low subtypes, exhibiting varying degrees of chemotherapy sensitivity [27, 28]. Thus, we first chose human TNBC MDA-MB-231 cells with the claudin-low subtype and MDA-MB-468 cells with the basal subtype, and established cells that express shRNA against Mint3 in a doxycycline (DOX)-inducible manner (ishMint3) (Fig. 1A, B) using these TNBC cell lines. Subsequently, the effect of Mint3 depletion on TNBC cell sensitivity to doxorubicin (DXR) and paclitaxel (PTX) was examined in vitro. Cytotoxicity assays showed no difference in sensitivity to DXR and PTX between the control (DOX−) and Mint3-depleted (DOX+) TNBC cells (Fig. 1C‒F). Thus, Mint3 is not involved in the chemoresistance of TNBC cells, at least in vitro.

### Mint3 depletion sensitizes TNBC cell lines to chemotherapy in vivo

**Fig. 2:**
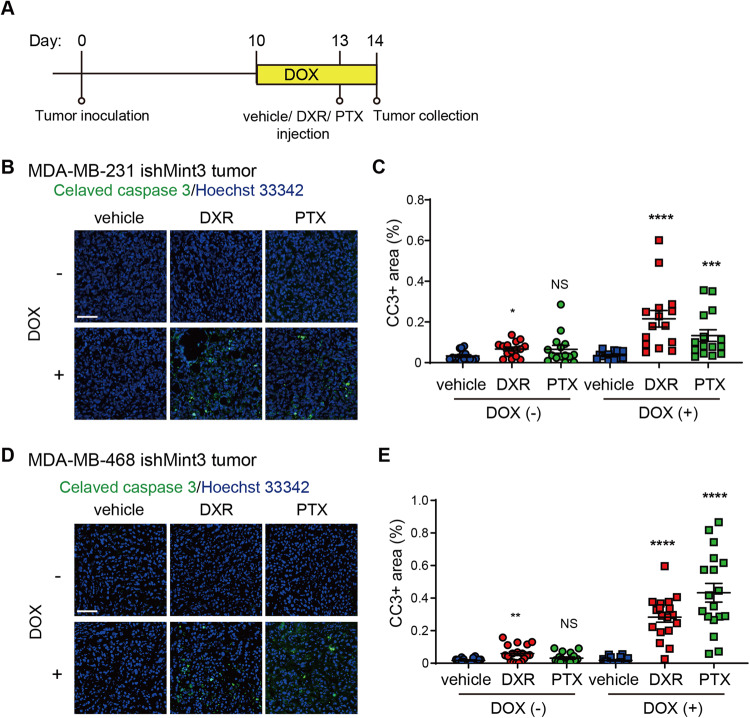
Mint3 depletion sensitizes TNBC cell lines to chemotherapy in vivo. **A** Schematic illustration of the chemotherapy-induced tumor apoptosis experiments. **B**–**E** Immunostaining for cleaved caspase-3 in tumors from control (DOX (−)) and Mint3-depleted (DOX (+)) MDA-MB-231 (**B**, **C**) and MDA-MB-468 (**D**, **E**) cells 24 h after chemotherapy. **B**, **D** Representative images are shown. Bar = 50 µm. **C**, **E** Cleaved caspase-3-positive areas were counted in tumor sections. *n* = 18 from six tumors per group. The data are presented as the mean ± SEM and were analyzed using the Mann–Whitney *U*-test. *p < 0.05, **p < 0.01, ****p < 0.0001. NS not significant.

**Fig. 3:**
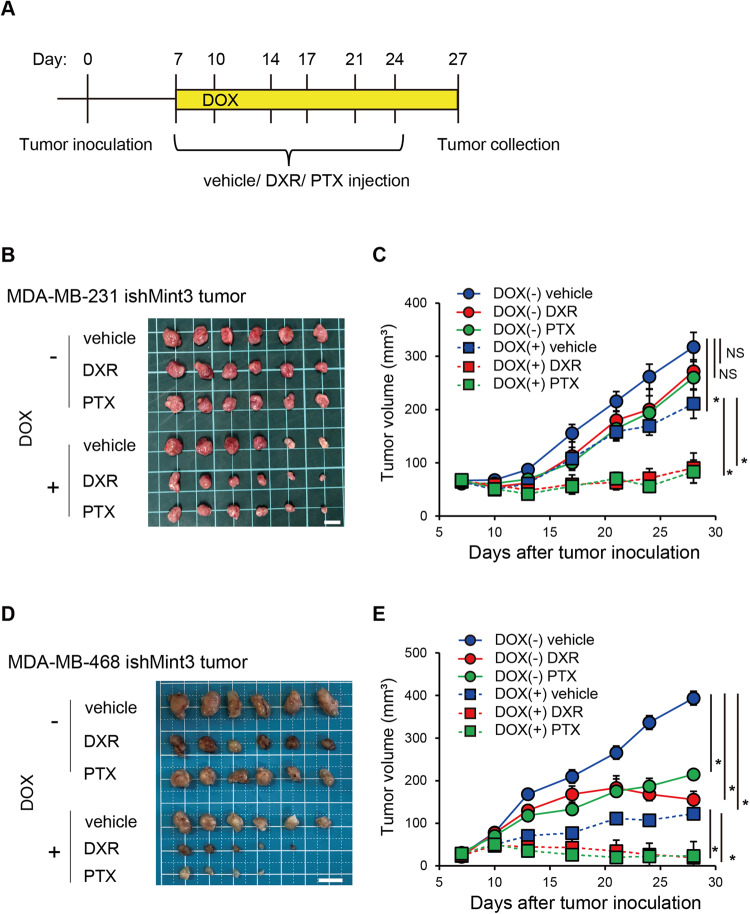
A combination of Mint3 depletion and chemotherapy suppresses tumor growth in TNBC cell lines. **A** Schematic illustration of the tumor growth assay. **B**–**E** Tumor growth of MDA-MB-231 ishMint3 (**B**, **C**) and MDA-MB-468 ishMint3 (**D**, **E**) cells in immunodeficient mice treated with or without doxycycline (DOX; 2 mg/mL in tap water) and vehicle, doxorubicin (DXR; 2 mg/kg bw), or paclitaxel (PTX; 20 mg/kg bw). **B**, **D** Images of tumors on day 28. Bar = 1 cm. **C**, **E** Tumor volume. *n* = 6 per group. The data are presented as the mean ± SEM and were analyzed using the Mann–Whitney *U*-test. *p < 0.05. NS not significant.

We next examined whether Mint3 depletion affects the chemoresistance of TNBC cells in vivo. To address this, MDA-MB-231 and MDA-MB-468 ishMint3 cells were inoculated into immunodeficient mice, and Mint3 expression in TNBC tumors was suppressed by DOX administration before chemotherapy (Fig. 2A). Intriguingly, DOX administration significantly increased the number of apoptotic cells in the tumors of MDA-MB-231 and MDA-MB-468 ishMint3 cells one day after DXR and PTX (Fig. 2B‒E and Supplementary Fig. S1A, B). DOX administration also increased the number of apoptotic cells in tumors of DOX-inducible Mint3 knockdown MDA-MB-231 cells with different shRNA sequences from ishMint3 (ishMint3#2) but not in those of control MDA-MB-231 (ishCTR) cells (Supplementary Fig. S1C‒H), indicating that Mint3 depletion increases apoptosis in TNBC tumors. These results prompted us to examine the combination therapy of Mint3 depletion and chemotherapy in the tumor growth of TNBC xenografts (Fig. 3A). DXR or PTX alone did not affect the tumor growth of MDA-MB-231 cells, whereas DOX-induced Mint3 depletion attenuated it, similar to the results of stably Mint3-depleted cells [21, 24] (Fig. 3B, C). Interestingly, the administration of DXR and PTX significantly attenuated the tumor growth of Mint3-depleted MDA-MB-231 ishMint3 cells (Fig. 3B, C). MDA-MB-468 cells were more sensitive to DXR and PTX than MDA-MB-231 cells, indicating that the basal subtype of breast cancer is more sensitive to chemotherapy than the claudin-low subtype [27, 28], and the combination of Mint3 depletion and chemotherapy strikingly reduced tumor volumes in these cells (Fig. 3D, E). Taken together, these results indicate that Mint3 depletion sensitizes TNBC cells to chemotherapy in vivo.

### Mint3 promotes HSP70 expression in TNBC in an in vivo-specific manner

**Fig. 4:**
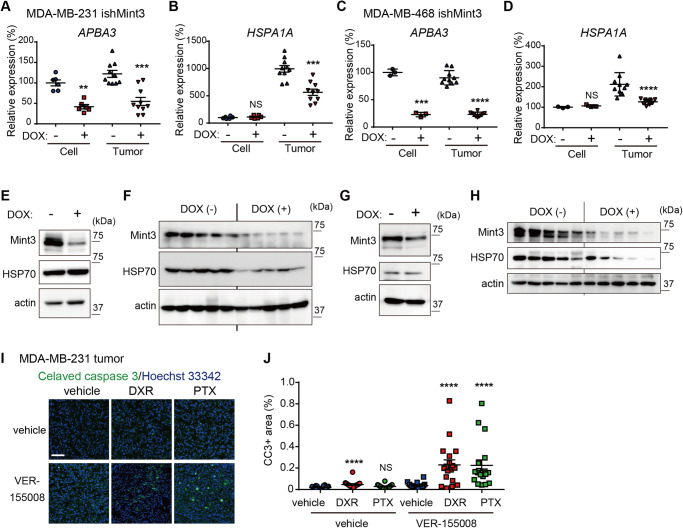
Mint3 promotes HSP70 expression in TNBC in an in vivo-specific manner. **A**–**D** mRNA levels of *APBA3* (encoding Mint3) (**A**, **C**) and *HSPA1A* (encoding HSP70) (**B**, **D**) in MDA-MB-231 (**A**, **B**) and MDA-MB-468 (**C**, **D**) ishMint3 cells and tumors treated with or without doxycycline (DOX). Data are presented as mean ± SEM and were analyzed using the Mann–Whitney *U*-test for MDA-MB-231 cells and MDA-MB-231 and MDA-MB-468 tumors, or the Welch’s *t*-test for MDA-MB-468 cells. **p < 0.01, ***p < 0.001, ****p < 0.0001. NS: not significant. **E**, **F** Protein levels of Mint3 and HSP70 in MDA-MB-231 ishMint3 cells (**E**) and tumors of these cells (*n* = 5 per group) (**F**) treated with or without doxycycline (DOX). **G**, **H** Protein levels of Mint3 and HSP70 in MDA-MB-468 ishMint3 cells (**G**) and tumors of these cells (*n* = 5 per group) (**H**) treated with or without doxycycline (DOX). **I**, **J** Immunostaining for cleaved caspase-3 in tumors from MDA-MB-231 cells 24 h after one-shot chemotherapy with the vehicle, DXR, or PTX in combination with the HSP70 inhibitor VER 155008 (25 mg/kg bw). **I** Representative images. **J** Cleaved caspase-3-positive areas were counted in the tumor sections. *n* = 18 from six tumors per group. The data are presented as the mean ± SEM and were analyzed using the Mann–Whitney *U*-test. ****p < 0.0001. NS not significant.

Mint3 depletion did not affect the chemoresistance of TNBC cells in vitro but attenuated it in vivo. Thus, we next addressed how Mint3 specifically promotes the chemoresistance of TNBC cells in vivo by analyzing the transcriptome of tumors from control and Mint3-depleted MDA-MB-231 cells (Supplementary Fig. S2A). We selected more than 1.5-fold overrepresented/underrepresented genes in the tumors of Mint3-depleted MDA-MB-231 cells as candidates that may be involved in Mint3-dependent chemoresistance. Under these criteria, eight genes were underrepresented and nine were overrepresented in tumors of Mint3-depleted MDA-MB-231 cells (Supplementary Fig. S2B, C). Among the underrepresented genes, we focused on *HSPA1A* which encodes HSP70 and has been reported to enhance chemoresistance [29–31]. We re-evaluated the mRNA levels of *APBA3*, which encodes Mint3, and *HSPA1A* in cultured cells and tumors of control and Mint3-depleted MDA-MB-231 and MDA-MB-468 cells. *HSAPA1A* mRNA levels were decreased by Mint3 depletion, specifically in the tumors of MDA-MB-231 and MDA-MB-468 cells (Fig. 4A‒D). In parallel to mRNA levels, HSP70 protein expression was also decreased by Mint3 depletion, specifically in the tumors of MDA-MB-231 and MDA-MB-468 cells (Fig. 4E‒H). The specificity of Mint3-mediated HSP70 expression in the tumors of MDA-MB-231 cells was also confirmed using control shRNA and another shRNA against Mint3 (Supplementary Fig. S2D, E). In addition, administration of naphthofluorescein, which inhibits Mint3-mediated HIF-1 activation, decreased HSP70 expression in the tumors of MDA-MB-231 cells (Supplementary Fig. S2F), reflecting a previous report that naphthofluorescein administration enhanced the antitumor effects of doxorubicin in tumors of MDA-MB-231 cells [26]. Subsequently, we examined whether HSP70 inhibition sensitized the tumors of MDA-MB-231 cells to the same extent as Mint3 depletion did in our experimental models. VER-15508 sensitized MDA-MB-231 cells to both DXR and PTX in vitro (Supplementary Fig. S3A, B). Furthermore, the concurrent administration of VER-155008, an HSP70 inhibitor, alongside DXR or PTX increased the number of apoptotic cells in MDA-MB-231 tumors when compared with chemotherapy alone (Fig. 4I). Conversely, exogenously expressed HSP70 in Mint3 knockdown induced chemoresistance to DXR and PTX both in vitro and in vivo (Supplementary Fig. 3C‒H). In summation, although HSP70 contributes to chemoresistance both in vitro and in vivo, it is noteworthy that Mint3 depletion decreases HSP70 expression and sensitizes TNBC cells to chemotherapy in an in vivo-specific manner.

### HIF-1 promotes HSP70 expression in TNBC in an in vivo-specific manner

**Fig. 5:**
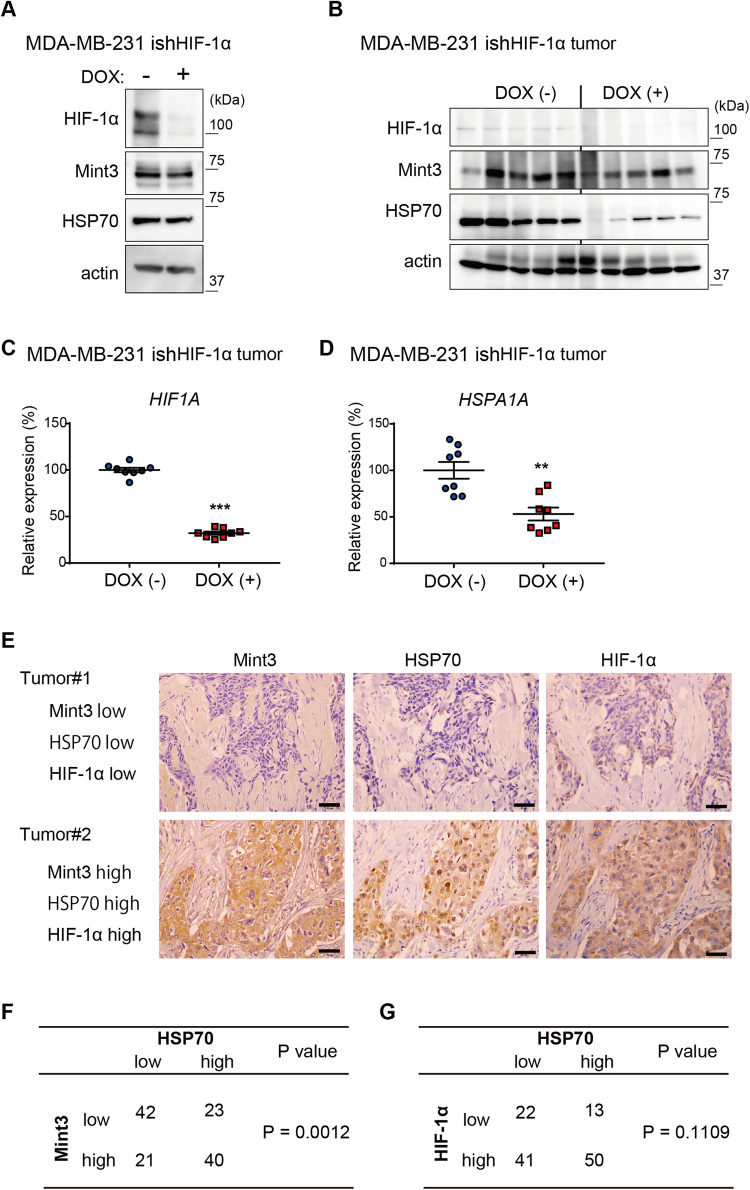
HIF-1 promotes HSP70 expression in TNBC in an in vivo-specific manner. **A** Protein level of HIF-1α, Mint3, and HSP70 in MDA-MB-231-expressing doxycycline-inducible shRNAs against HIF-1α (ishHIF-1α) cells treated with or without doxycycline (DOX; 1 μg/ml) for three days. **B** Protein levels of HIF-1α, Mint3, and HSP70 in MDA-MB-231 ishHIF-1α tumors administered with or without DOX (2 mg/mL in tap water) for 3 days. *n* = 5 per group. **C**, **D** mRNA levels of *HSPA1A* (**C**) and *HIF1A* (**D**) in MDA-MB-231 ishHIF-1α tumors administered with or without doxycycline for 3 days. *n* = 8 per group. Data represent mean ± SEM and were analyzed using the Mann–Whitney *U*-test. **p < 0.01, ***p < 0.001. **E**–**G** Immunostaining for Mint3, HIF-1α, and HSP70 in TNBC specimens (*n* = 126). **E** Representative immunostaining images. Bar = 50 µm. **F** The correlation between Mint3 and HSP70 protein expression was statistically analyzed using the chi-square test. **G** The correlation between HIF-1α and HSP70 protein expression was statistically analyzed using Fisher’s exact test.

Mint3 activates HIF-1 transcriptional activity by suppressing FIH-1 [22, 23]. Thus, next, we evaluated whether HIF-1α depletion decreased HSP70 expression in MDA-MB-231 tumors, similar to Mint3 depletion. To address this, we established DOX-inducible HIF-1α knockdown MDA-MB-231 cells (ishHIF-1α) (Fig. 5A) and evaluated HSP70 expression in cultured cells and tumors. HIF-1α depletion did not affect Mint3 expression in either cultured cells or tumors of MDA-MB-231 cells, whereas HSP70 protein levels decreased in the tumors of MDA-MB-231 cells when HIF-1α was depleted (Fig. 5A, B). mRNA levels of *HSPA1A* also decreased in the tumors of HIF-1α-depleted MDA-MB-231 cells (Fig. 5C, D). Thus, HIF-1 also promoted HSP70 expression in an in vivo-specific manner in MDA-MB-231 cells. Next, we examined whether the Mint3-HIF-1 axis promotes HSP70 expression in clinical TNBC specimens. Immunostaining analysis of TNBC tissue microarrays showed that Mint3 expression was positively correlated with HSP70 expression, whereas there was no significant correlation between HIF-1α and HSP70 expression in TNBC specimens (Fig. 5E‒G). These results suggest that HIF-1 expression is a prerequisite, but Mint3-mediated activation of HIF-1 is essential for HSP70 induction in TNBC.

### Mint3 activates HSF-1 via the mechanistic target of the rapamycin (mTOR) signaling pathway in TNBC tumors

**Fig. 6:**
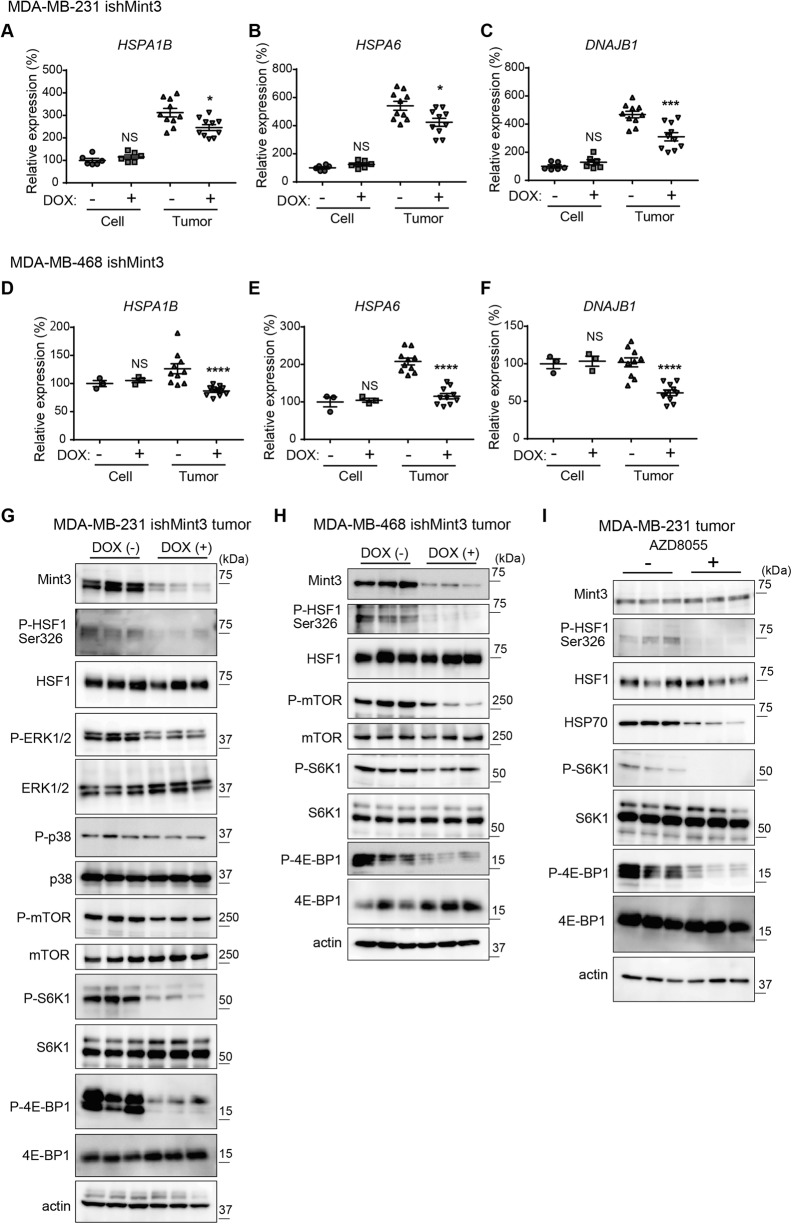
Mint3 activates HSF-1 via the mTOR signaling pathway in TNBC tumors. **A**–**F** mRNA levels of HSF1 target genes (*HSPA1B*, *HSPA6*, and *DNAJB1*) in MDA-MB-231 (**A**–**C**) and MDA-MB-468 (**D**–**F**) ishMint3 cells and tumors treated with or without doxycycline (DOX). Data are presented as mean ± SEM and were analyzed using the Mann–Whitney U-test for MDA-MB-231 cells and MDA-MB-231 and MDA-MB-468 tumors, or the Welch’s *t*-test for MDA-MB-468 cells. *p < 0.05, ***p < 0.001, ****p < 0.0001. NS: not significant. **G** Immunoblotting of HSF-1 and its upstream signaling-related proteins in MDA-MB-231 ishMint3 tumors treated with or without DOX for three days. *n* = 3 per group. **H** Immunoblotting of HSF-1 and mTOR signaling-related proteins in MDA-MB-468 ishMint3 tumors treated with or without DOX for 3 days. *n* = 3 per group. **I** Immunoblotting of Mint3, HSF-1, and mTOR signaling-related proteins in MDA-MB-231 tumors treated with or without the mTOR inhibitor AZD8055 (10 mg/kg) for 3 days. *n* = 3 per group.

Subsequently, we addressed the mechanisms of how the Mint3–HIF-1 axis induces HSP70 expression in an in vivo-specific manner in TNBC. We first hypothesized that hypoxic conditions observed in tumors may trigger HSP70 induction via the Mint3–HIF-1 axis in TNBC. However, hypoxia alone could not recapitulate the in vivo-specific HSP70 induction by the Mint3–HIF-1 axis in MDA-MB-231 cells (Supplementary Fig. S4A, B). The JASPAR CORE database (https://jaspar.genereg.net/) showed a hypoxia response element ~448–444 bp upstream of the transcription-stating site of *HSPA1A*. However, chromatin immunoprecipitation assays using anti-HIF-1α antibodies showed that HIF-1 accumulated at the locus of known HIF-1 target genes, but not at the *HSPA1A* locus (Supplementary Fig. S4C), indicating that *HSPA1A* was not a direct target of HIF-1, even in MDA-MB-231 cells. HSF1 is a master transcription factor for heat shock proteins, including HSP70 [4, 5, 32]. We could not identify HSF-1 target genes, except *HSPA1A* in the RNA-seq analyses of tumors of Mint3-depleted MDA-MB-231 cells, probably because of the small sample size and great variation in expression levels (Supplementary Fig. S2B, C). However, we re-evaluated the mRNA levels of HSF1 target genes such as *HSPA1B*, *HSPA6*, and *DNAJB1* in cultured cells and tumors of MDA-MB-231 and MDA-MB-468 cells using RT-qPCR. Mint3 depletion decreased mRNA levels of HSF1 target genes in tumors but not in cultured MDA-MB-231 and MDA-MB-468 cells, although the decrease in expression levels was moderate compared to that of *HSPA1A* (Fig. 6A‒F). HIF-1α depletion also decreased the mRNA levels of HSF1 target genes in MDA-MB-231 cells (Supplementary Fig. S5A‒C). These results prompted us to analyze how Mint3 controls HSF1 expression in TNBC. HSF1 activity is regulated by various post-translational modifications, among which, phosphorylation at the Ser326 residue of HSF1 stands as a well-established and extensively characterized post-translational modification for its activation [4, 5, 32]. Thus, we examined the levels of HSF1 phosphorylation at Ser326 (p-HSF1 Ser326) in the tumors of MDA-MB-231 cells. Surprisingly, Mint3 depletion attenuated p-HSF1 Ser326 levels in tumors of MDA-MB-231 cells. Extracellular signal-regulated kinase, p38, and mTOR phosphorylate HSF1 Ser326 [4, 5]. Mint3 depletion slightly decreased the phosphorylation of extracellular signal-regulated kinase 1/2 and mTOR Ser2448, but not p38, in MDA-MB-231 tumors (Fig. 6G). Interestingly, the phosphorylation levels of two representative mTOR targets, S6K1 and 4E-BP1, were strikingly attenuated in tumors of Mint3-depleted MDA-MB-231 cells (Fig. 6G), indicating that mTOR activity was suppressed in tumors of such cells. Similar to MDA-MB-231 cells, Mint3-depleted MDA-MB-468 tumors showed decreased levels of p-HSF1 Ser326 and mTOR activity (Fig. 6H). We then examined whether decreased mTOR activity indeed attenuated p-HSF1 Ser326 levels and HSP70 expression in MDA-MB-231 tumors. Administration of the mTOR inhibitor AZD8055 attenuated mTOR activity, p-HSF1 Ser326 levels, and HSP70 expression in MDA-MB-231 tumors (Fig. 6I). Thus, Mint3-depletion-mediated mTOR inactivation attenuates HSF1 activity, resulting in the decreased expression of HSF1 target genes, including HSP70, in TNBC tumors.

### Mint3 depletion induces energy stress and thereby attenuates HSF-1 activity in TNBC tumors

**Fig. 7:**
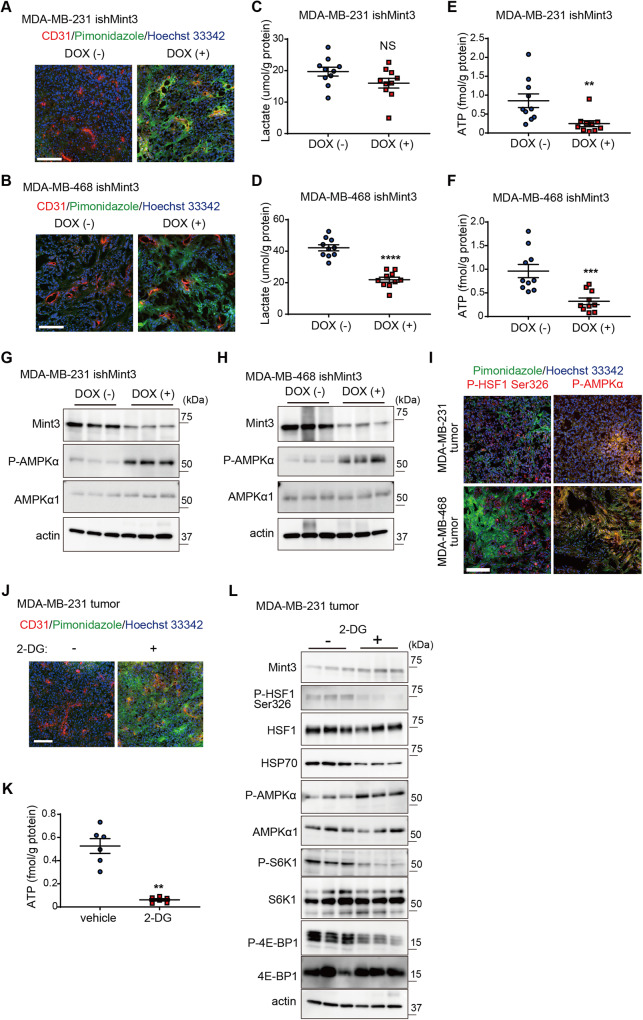
Mint3 depletion induces energy stress and thereby attenuates HSF-1 activity in TNBC tumors. **A**, **B** Immunostaining of hypoxic markers pimonidazole (green) and endothelial cell marker CD31 (red) in MDA-MB-231 (**A**) and MDA-MB-468 (**B**) ishMint3 tumors administered with or without doxycycline (DOX) for 3 days. Nuclei were counterstained with Hoechst 33342 (blue). **C**, **D** Lactate levels in MDA-MB-231 (**C**) and MDA-MB-468 (**D**) ishMint3 tumors administered with or without DOX for three days. *n* = 10 per group. **E**, **F** ATP levels in MDA-MB-231 (**E**) and MDA-MB-468 (**F**) ishMint3 tumors administered with or without DOX for 3 days. *n* = 10 per group. **G**, **H** Phosphorylation levels of AMPK1α in MDA-MB-231 (**G**) and MDA-MB-468 (**H**) ishMint3 tumors administered with or without DOX for 3 days. *n* = 3 per group. **I** Immunostaining of hypoxic marker pimonidazole (green) and phospho-AMPK1α or phospho-HSF-1 Ser326 (red) in MDA-MB-231 and MDA-MB-468 tumors. Nuclei were counterstained with Hoechst 33342 (blue). **J** Immunostaining of hypoxic marker pimonidazole (green) and endothelial cell marker CD31 (red) in MDA-MB-231 tumors administered with or without a glycolysis inhibitor 2-deoxyglucose (2-DG; 500 mg/kg b.w.) for 3 days. Nuclei were counterstained with Hoechst 33342 (blue). **K** ATP levels in MDA-MB-231 tumors were administered with or without 2-DG for 3 days. *n* = 6 per group. **L** Immunoblotting of Mint3, HSF-1, HSP70, and mTOR signaling-related proteins in MDA-MB-231 tumors administered with or without 2-DG for 3 days. *n* = 3 per group. In **C**–**F** and **K**, the data represent the mean ± SEM and were analyzed using the Mann–Whitney *U*-test. **p < 0.01, ***p < 0.001, ****p < 0.0001. NS not significant.

Finally, we addressed how Mint3 depletion attenuates mTOR activity in TNBC tumors. Mint3 activates HIF-1, thereby enhancing glycolytic activity even during normoxia in cancer cells [21, 23]. Enhanced glycolytic activity in cancer cells is known as the Warburg effect [15]. The Warburg effect is thought to provide cancer cells with advantages such as rapid ATP production via glycolysis and metabolic by-products used for cell proliferation [16–18, 33]. Under tumor conditions, the Warburg effect is also proposed to reduce oxygen consumption via mitochondrial ATP production, thereby avoiding severe hypoxia [16]. Thus, we examined hypoxic areas in control and Mint3-depleted TNBC tumors using pimonidazole, whose adducts can be detected in areas with severe hypoxia (*p*O_2_ < 10 mm Hg) [34]. Surprisingly, Mint3 depletion caused severe hypoxia, even in the blood vessel-rich areas of MDA-MB-231 and MDA-MB-468 tumors (Fig. 7A, B) but not in vitro (Supplementary Fig. S6A, B). Despite more hypoxic areas, lactate levels were not elevated in Mint3-depleted MDA-MB-231 tumors (Fig. 7C) but were decreased in Mint3-depleted MDA-MB-468 tumors (Fig. 7D). Reflecting this metabolic maladaptation to hypoxic conditions, ATP levels were significantly decreased in the tumors of Mint3-depleted TNBC cells (Fig. 7E, F). Decreased ATP levels activate adenosine monophosphate-activated protein kinase α (AMPKα), which suppresses mTOR activity [35, 36]. Corresponding to decreased ATP levels, Mint3 depletion promoted phosphorylation of AMPKα, indicating AMPKα activity in tumors of MDA-MB-231 and MDA-MB-468 cells (Fig. 7G, H). The administration of metformin, an AMPK activator, also reduced HSP-1 expression. This was accompanied by a decrease in the phosphorylation levels of S6K1, 4E-BP1, and HSF-1 Ser326 within the tumors of MDA-MB-231 cells, mirroring the effects observed with Mint3 depletion (Supplementary Fig. S7A). Notably, the phosphorylation levels of AMPK1 were elevated in response to Mint3 depletion, despite the reduction in HSP-70, even in the context of TNBC tumors grown within the mammary fat pad (Supplementary Fig. S7B, C). However, it is crucial to underline that this Mint3 depletion-mediated AMPK activation was not evident in vitro (Supplementary Figs. S7D and E). Immunostaining showed that p-HSF1 Ser326-positive cells existed mainly in non-hypoxic areas, whereas phosphorylated AMPKα-positive cells existed mainly in hypoxic areas (Fig. 7I), indicating that hypoxia activates AMPK activity and inactivates HSF1 activity in TNBC tumors. Finally, we examined whether glycolysis inhibition phenocopies Mint3 depletion in MDA-MB-231 tumors. Administration of the glycolysis inhibitor 2-deoxy-d-glucose (2-DG) induced hypoxia in the blood vessel-rich areas of MDA-MB-231 tumors (Fig. 7J). In addition, 2-DG administration decreased ATP levels, activated AMPK1, inactivated mTOR, and decreased p-HSF1 Ser326 and HSP70 in MDA-MB-231 tumors, similar to Mint3 depletion (Fig. 7K, L). In turn, 2-DG partially decreased the ATP production in MDA-MB-231 cells in vitro, resulting in comparable AMPK phosphorylation and HSP70 expression levels in 2-DG-treated cells (Supplementary Fig. S8A, B). Thus, defects in glycolysis induce energy stress and thereby attenuate HSF-1 activity in Mint3-depleted TNBC tumors.

## Discussion

**Fig. 8:**
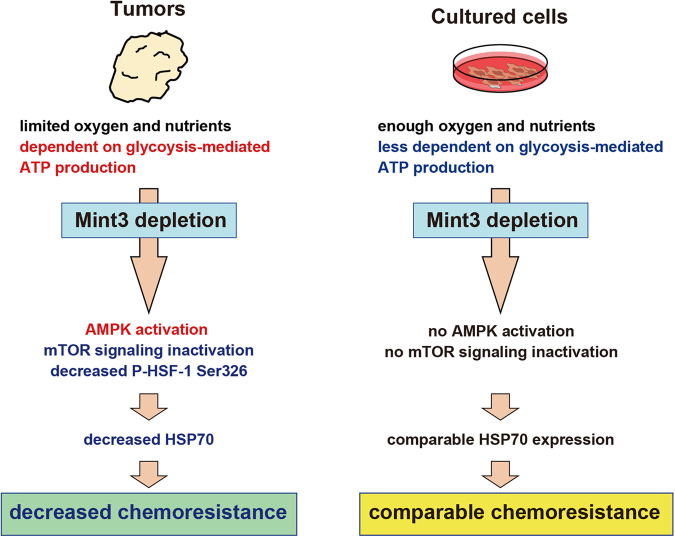
Illustration of the role of Mint3 in promoting chemoresistance in TNBC tumors. TNBC predominantly relies on glycolysis-mediated ATP production in tumors with limited oxygen and nutrients. Remarkably, Mint3 depletion affects glycolysis-mediated ATP production and activates AMPK, resulting in decreased HSF-1 Ser326 phosphorylation and HSP70 expression. The ensuing reduction in HSP70 levels sensitizes TNBC tumors to chemotherapy. Conversely, TNBC cells cultured under conditions of ample oxygen and nutrient availability exhibit a reduced dependency on glycolysis-mediated ATP production. Consequently, Mint3 depletion fails to exert an impact on AMPK activity or HSP70 expression, resulting in comparable chemoresistance in TNBC cells.

Although the detailed mechanisms of chemoresistance have been extensively studied in cultured cells, the mechanism by which TNBC resists chemotherapy in vivo remains unclear. Here, we showed that metabolic adaptation to the tumor microenvironment via Mint3 contributes to chemoresistance in TNBC. The Mint3–HIF-1 axis activates glycolysis and energy production, activating mTOR. Activated mTOR phosphorylates HSF-1 at Ser326, thereby activating HSF-1. Activated HSF-1 promoted HSP70 expression, which contributed to chemoresistance in TNBC (Fig. 8). It is well documented in the scientific literature that both 2-DG and metformin sensitize tumors to chemotherapy [37–43]. These results collectively suggest a possible link between HSP70 expression, facilitated via glycolysis-mediated ATP production regulated by Mint3 in tumors, and the previously reported chemosensitizing effects of 2-DG and metformin, at least in part. Although administration of the HSP70 inhibitor VER-155008 enhanced the efficacy of doxorubicin and paclitaxel in MDA-MB-231 and MDA-MB-468 tumors, we cannot exclude the possibility that other HSF-1 target genes also contribute to the chemoresistance of TNBC in vivo. Furthermore, while Mint3 depletion sensitized cancer cells to chemotherapy via HSP70 reduction, other mechanisms such as reduced VEGFA production [21, 25] and physical characteristics such as reduced tumor size may further contribute to the enhanced efficacy of chemotherapy in Mint3-depleted tumors.

Elevated glycolysis in cancer cells during normoxia is known as the Warburg effect [15]. Many studies have shown that depleting elevated glycolytic enzymes can attenuate tumor growth in various types of cancer [11–14]. Indeed, the Warburg effect must be involved in these phenotypes. However, the depletion of glycolysis-related genes affects not only the Warburg effect but also the Pasteur effect, which elevates glycolysis during hypoxia [33]. Thus, the precise role of the Warburg effect in vivo remains unclear. Mint3 activates HIF-1 during normoxia by inhibiting the oxygen-dependent HIF-1 suppressor, FIH-1 [22, 23]. Knockdown experiments using siRNA showed that Mint3 depletion attenuated lactate production in various cancer cell lines during normoxia but not during hypoxia [21], indicating the Warburg-effect-specific Mint3 contribution. Thus, attenuation of tumor growth and chemoresistance in TNBC tumors by Mint3 depletion might shed light on the role of the Warburg effect in vivo.

Here, we focused on the chemoresistance of TNBC and showed that Mint3 promotes the chemoresistance of TNBC tumors by activating HSF-1 and inducing HSP70 expression. HSF-1 is a master regulator of heat shock proteins. However, independent of the heat shock response, HSF-1 has been reported to support malignant features such as cell cycle regulation, signaling, metabolism, adhesion, and translation in various cancers including breast cancer [32]. Thus, Mint3-mediated HSF-1 activation may also contribute to various malignant features independent of the heat shock response in TNBC. Although both HSF-1 and HIF-1 are essential transcription factors for stress responses, a limited number of studies have reported that HSF family proteins, HSF2 and HSF4, promote transcription of HIF-1α and result in *VEGFA* expression in several cancer cell lines [44]. Our findings provide novel crosstalk between HIF-1 and HSF-1 mediated by Mint3 in tumor microenvironments and shed light on the strategy whereby TNBC resists chemotherapy by integrating multiple stress responses. Although Mint3 can activate HIF-1 both in vitro and in vivo, HSF-1 inactivation and attenuation of chemoresistance by Mint3 depletion were observed only in vivo. One of the reasons for this is that attenuated glycolysis by Mint3 depletion did not have a significant impact on the energy production of cancer cells under nutrient-rich culture conditions. In addition, various stress-induced post-translational modifications are necessary for HSF-1 to be fully activated [4, 5]. Normal cell culture conditions do not seem to match such stressful conditions sufficiently to activate HSF-1. Taken together, Mint3 depletion did not affect HSP70 expression or chemoresistance in TNBC under culture conditions. It is imperative to acknowledge a certain limitation of our study. We conducted an analysis of whole tumor gene expression via RNA-seq and RT-qPCR, revealing only modest alterations induced by Mint3 depletion. Considering that local oxygen concentration can impact the consequences of Mint3 depletion, we posit that the utilization of single-cell RNA-seq analysis may reveal the impact of Mint3 depletion in tumors more clearly than bulk RNA-seq analysis.

In summary, Mint3 depletion induces metabolic inadaptation to the tumor microenvironment, thereby sensitizing TNBC to chemotherapy in vivo. The combination of Mint3 inhibition and chemotherapy may be a good strategy for TNBC treatment.

## Materials and methods

Detailed information on material and methods is available in Supplementary Information and Supplementary Tables 1 and 2. Full-length, uncropped original western blots are available in the Supplementary File.

### Supplementary information

SUPPLEMENTARY MATERIALS AND METHODS

Supplementary Fig. S1

Supplementary Fig. S2

Supplementary Fig. S3

Supplementary Fig. S4

Supplementary Fig. S5

Supplementary Fig. S6

Supplementary Fig. S7

Supplementary Fig. S8

Supplementary Table S1

Supplementary Table S2

original data

## Data Availability

Data supporting the findings of this study are included in the figures and supporting files.
