# Supplementary material for: Mint3-depletion-induced energy stress sensitizes triple-negative breast cancer to chemotherapy via HSF1 inactivation
Source: Cell Death Dis. 2023 Dec 11;14(12):815. doi: 10.1038/s41419-023-06352-4 (PMC10713533; doi:10.1038/s41419-023-06352-4)
Supplement: Supplementary file 1 — SUPPLEMENTARY MATERIALS AND METHODS [file 41419_2023_6352_MOESM1_ESM.docx]

**Supplementary Information**

**Mint3-depletion-induced energy stress sensitizes triple-negative breast cancer to chemotherapy via HSF1 inactivation**

**Noritaka Tanaka^1#^, Hikari Okada^2#^, Kiyoshi Yamaguchi^3^, Masahide Seki^4^, Daisuke Matsubara^5^, Noriko Gotoh^6^, Yutaka Suzuki^4^, Yoichi Furukawa^3^, Taro Yamashita^7^, Jun-ichiro Inoue^8^, Shuichi Kaneko^2^, and Takeharu Sakamoto^1, 7*^**

^1^ Department of Cancer Biology, Institute of Biomedical Science, Kansai Medical University, Osaka, Japan.

^2^ Information-Based Medicine Development, Graduate School of Medical Sciences, Kanazawa University, Ishikawa, Japan.

^3^ Division of Clinical Genome Research, the Institute of Medical Science, The University of Tokyo, Tokyo, Japan.

^4^ Department of Computational Biology and Medical Sciences, Graduate School of Frontier Sciences, the University of Tokyo, Chiba, Japan.

^5^ Department of Pathology, University of Tsukuba, Ibaraki, Japan.

^6^ Division of Cancer Cell Biology, Cancer Research Institute, Kanazawa University, Ishikawa, Japan.

^7^ Department of System Biology, Institute of Medical, Pharmaceutical and Health Sciences, Kanazawa University, Ishikawa, Japan.

^8^ The University of Tokyo Pandemic Preparedness, Infection and Advanced Research Center (UTOPIA), Tokyo, Japan.

**#These authors contributed equally to this work.**

***Address correspondence to:** Takeharu Sakamoto, Ph.D., Department of Cancer Biology, Institute of Biomedical Science, Kansai Medical University, 2-5-1 Shin-machi, Hirakata, Osaka 573-1010, Japan. Tel: +81-72-804-2363; E-mail: sakamott@hirakata.kmu.ac.jp

**SUPPLEMENTARY Materials and Methods**

**Cell culture**

MDA-MB-231 and MDA-MB-468 cells were obtained from the American Type Culture Collection (Manassas, VA, USA). Cells were cultured in Dulbecco’s modified Eagle’s medium containing 10% fetal bovine serum, 100 units/mL penicillin, and 100 µg/mL streptomycin at 37°C in a humidified incubator with 5% CO_2_. For experiments conducted under hypoxic conditions, cells were cultured with 1% O_2_ and 5% CO_2_ in a Model 9200 incubator (Wakenyaku, Kyoto, Japan) for 24 h as previously described [1]. Doxycycline-inducible shRNA-expressing cells were prepared using lentiviral vectors (SMART vector) purchased from Horizon (Waterbeach, UK). The catalog numbers of the lentiviral vectors were as follows: ishMint3, V3IHSMCG_5384378; ishMint3#2, V3IHSMCG_6928646, ishHIF-1α, and V3IHSMCG_5522681. The shRNA vector designed for *APBA3* mRNA (which encodes Mint3) but did not affect Mint3 expression (V3IHSMCG_6286829), was used as a control vector (ishCTR). All the cell lines were routinely tested to exclude mycoplasma contamination.

**Lentiviral HSAPA1A expression vector construction**

The human *HSAPA1A* cDNA was amplified from mouse brain tissue of the MDA-MB-231 cell line through RT-PCR. Subsequently, it was subcloned into the pENTR/D-TOPO vector (Thermo Fisher Scientific, Waltham, MA, USA) before being incorporated into the lentivirus vector pLenti6/V5 DEST (Thermo Fisher Scientific), as previously described [2]. The lentiviral vectors were generated and used according to the manufacturer’s instructions, followed by their transfection into MDA-MB-231 ishMint3#1 cells.

**Reagents**

Doxycycline and Hoechst 33342 were purchased from Sigma–Aldrich (St. Louis, MO, USA). Doxorubicin, paclitaxel, and 2-DG were purchased from FUJIFILM Wako (Osaka, Japan). VER-155008 was purchased from Cayman Chemical Co.(Ann Arbor, MI, USA). AZD8055 was purchased from Selleck Chemicals (Houston, TX, USA). Naphthofluorescein was synthesized by Sundia MediTech Company (Shanghai, China) at a purity > 95%.

**Tumor transplantation**

The experimental protocols were approved by the Animal Care and Use Committees of the Institute of Medical Science, University of Tokyo, the Institute of Medical, Pharmaceutical, and Health Sciences, Kanazawa University, and Kansai Medical University. They were also conducted following the institutional ethical guidelines for animal experiments and the safety guidelines for gene manipulation experiments. The sample size was based on the statistical analysis of variance and exploratory experiments. BALB/c nude mice were purchased from CLEA Japan (Tokyo, Japan) and maintained under specific pathogen-free conditions. Eight-week-old female BALB/c nude mice were used for the tumor growth assay via subcutaneous injection of tumor cells. For the growth studies, cells (1 × 10^6^) in 0.2 mL PBS were injected into the left flank of mice. On days 7 or 10, the mice were randomly assigned to each experimental group. The mice drank tap water or doxycycline-containing tap water (2 mg/mL) *ad libitum*. For the chemotherapy models, vehicle or anticancer reagents (doxorubicin 2 mg/kg b.w.; paclitaxel 20 mg/kg b.w.) were intraperitoneally injected into tumor-bearing mice twice a week. The implanted tumors were measured with calipers on the indicated days, and their volumes were calculated using the formula V = (L × W^2^)/2, where V is the volume (mm^3^), L is the largest tumor diameter (mm), and W is the smallest tumor diameter (mm). 2-DG (500 mg/kg b.w.) was intraperitoneally injected, and AZD8055 (10 mg/kg b.w. in 10% Kolliphor EL [Sigma–Aldrich]/saline) was orally administered to tumor-bearing mice for three days. Metformin (2 mg/mL in drinking water) was administrated to tumor-bearing mice for three days. Naphthofluorescein (100 mg/kg b.w. in saline containing 1% Kolliphor EL [Sigma–Aldrich] and 8 mM Na_2_CO_3_) was intraperitoneally administered to tumor-bearing mice for three days. For orthotopic injection into the fourth mammary fat pad, 1 × 10^6^ TNBC cells were suspended in 50 µL of PBS and mixed with 50 µL of Matrigel (Corning, Corning, NY, USA). Mice were anesthetized by butorphanol (Meiji Seika, Tokyo, Japan), medetomidine (Fujita Pharmaceutical company, Tokyo, Japan), and midazolam (Sandoz, Tokyo, Japan), and mammary tissues were accessed by midventral incision. The skin was detached without damage to the peritoneum and TNBC cells were injected without leakage. To detect hypoxic areas in the tumors, mice were intravenously injected with pimonidazole (100 mg/kg b.w.; Hypoxyprobe, Burlington, MA, USA) 30 min before sacrifice. All mice were included in the analysis. Investigators were not blinded during data acquisition and analysis.

**Western blotting analysis**

Cells were lysed with lysis buffer (1% NP-40, 50 mM Tris (pH 8.0), 150 mM NaCl) and centrifuged at 20,000 × *g* for 15 min at 4°C. Tumors were lysed in RIPA buffer (FUJIFILM Wako) containing protease inhibitors (Roche, Basel, Switzerland) and phosphatase inhibitors (Roche) by homogenization using TissueLyser II (Qiagen, Hilden, Germany) and centrifuged at 20,000 × *g* for 30 min at 4°C. The supernatants were collected, and the total protein content was measured using the Bradford assay (Bio-Rad, Hercules, CA, USA). Lysates were separated by sodium dodecyl sulfate-polyacrylamide gel electrophoresis, transferred to polyvinylidene fluoride membrane filters, and analyzed by western blotting. Detailed information regarding the antibodies used in this study is provided in Supplementary Table S1.

**Cytotoxicity assay**

Cells were seeded in 96-well plates at a density of 1 × 10^4^ cells/well. The next day, cells were treated with fresh doxorubicin or paclitaxel at the indicated concentrations for 24 h. Cell viability was determined using the Cell Counting Kit-8 (Dojindo, Kumamoto, Japan) according to the manufacturer’s instructions.

**RNA isolation, reverse transcription, and quantitative polymerase chain reaction**

Total RNA was isolated from cells or tumors using the RNeasy Plus Mini Kit (Qiagen) and subjected to reverse transcription (RT) using the ReverTra Ace qPCR RT Master Mix (TOYOBO, Osaka, Japan). The RT products were analyzed by real-time PCR in a 7500 real-time PCR system (Thermo Fisher Scientific, Waltham, MA, USA) using KOD SYBR qPCR (TOYOBO) and specific primers (Supplementary Table S2) as previously described [2, 3]. The expression levels of individual mRNAs were normalized to those of *ACTB* mRNA.

**Immunostaining**

Frozen sections of tumor tissues were prepared and subjected to immunostaining using specific antibodies (Supplementary Table S1) as previously described [1, 4, 5]. The positive areas of the indicated antibodies were quantified using the ImageJ software (National Institutes of Health, Bethesda, MD, USA).

**RNA-seq analysis**

Total RNA was isolated from the tumors of control (DOX(-)) and Mint3-depleted (DOX(+)) MDA-MB-231 cells using the RNeasy mini plus kit (Qiagen). RNA-seq libraries were prepared using the TruSeq Stranded mRNA Library Prep Kit (Illumina, San Diego, CA, USA), according to the manufacturer’s protocol. The libraries were sequenced with 50/49 bp pair-end reads on NovaSeq6000 (Illumina). Sequencing data were analyzed using a standard RNA-seq analytical pipeline. After removing contaminating mouse sequences using the Xenome tool (v1.0.0) [6], the sequencing reads were aligned to the human genome (hg38) using the STAR aligner (v2.7.3a) [7]. RSEM (v1.3.3) [8] was used to obtain raw gene counts from the read alignments. The DESeq2 package (v1.26.0) [9] was used to normalize the read count data and test for differential gene expression. Genes with a Benjamini–Hochberg adjusted p-value < 0.05 and more than 1.5-fold overrepresented/underrepresented in Mint3-depleted tumors were considered significantly different. RNA-seq data were deposited in the DDBJ databases (https://www.ddbj.nig.ac.jp/index-e.html) (Accession number: DRA016246).

**Chromatin immunoprecipitation (ChIP)-seq analysis**

Tumors were digested using the gentleMACS Dissociator and Tumor dissociation kit (Miltenyi Biotec, Bergisch Gladbach, Germany), and mouse stromal cells were removed using the Mouse Cell Depletion Kit (Miltenyi Biotec). Tumor cells were subjected to ChIP using the SimpleChIP Enzymatic Chromatin IP Kit (Cell Signaling Technology) and a rabbit anti-HIF-1α antibody (ab2185; Abcam, Cambridge, UK). After precipitation, 1 ng of DNA and the NEBNext Ultra II DNA Library Prep kit (New England Biolabs, Ipswich, MA, USA) were used to prepare libraries according to the manufacturer's instructions. The libraries were sequenced with 60-bp single-end reads on the HiSeq2500 platform (Illumina). Raw sequencing reads were analyzed for quality using FastQC and then aligned to the human genome (GRCh38) using Bowtie2 (v2.4.1) [10]. Peak calling, followed by the assignment of peaks to genes, was performed using MACS2 (v3.6) [11] and HOMER (v4.11) [12]. Peaks with a q-value < 0.05 were considered significant. ChIP-seq data were deposited in the DDBJ databases (https://www.ddbj.nig.ac.jp/index-e.html) (Accession number: DRA016247).

**Tissue microarray analysis**

Immunohistochemical staining was performed using a paraffin-embedded pancreatic cancer tissue array purchased from US BIOMAX (BR1301; Derwood, MD, USA). The sections were deparaffinized in xylene and rehydrated in a graded ethanol series. Antigen retrieval was carried out for HSP70 immunostaining by autoclaving in 10 mM citrate buffer (pH 6.0) for 10 min at 121°C, followed by cooling for 20 min. After blocking endogenous peroxidase activity with a 3% aqueous H_2_O_2_ solution for 5 min, the sections were incubated with mouse monoclonal anti-HSP70 (1:50; 386032; Merck Millipore, Burlington, MA, USA), mouse monoclonal anti-human Mint3 (1:100; #611380; BD Biosciences, Franklin Lakes, NJ, USA), and rabbit anti-HIF-1α (1:100; #NB100-479; Novus Biologicals, Centennial, CO, USA) antibodies at 4°C overnight. After washing with TBS, HSP70, Mint3, and HIF-1α antibodies were detected using the DAKO Envision+ Dual Link System. 3,3ʹ-Diaminobenzidine tetrahydrochloride was used as a chromogen, and hematoxylin was used as a light counterstain. Evaluation of immunohistochemical staining via light microscopy was performed by a pathologist (D. M.) who was blinded to the clinical data of each patient. The immunoreactivity of HSP70, Mint3, and HIF-1α was evaluated semi-quantitatively based on the intensity and estimated percentage of tumor cells stained as previously described [1]. Briefly, the intensity was quantified as follows: 1+, weak staining (detection required high magnification); 2+, moderate staining (detected readily at medium magnification); 3+, strong staining (detected readily at low magnification). The percentages of positive cells were scored into five categories: 0, 0%; 1, 1–25%; 2, 26–50%; 3, 51–75%; 4, 76–100%. The product of intensity and percentage scores was used as the final staining score. The final scores for Mint3, HSP70, and HIF-1α were defined as low-level expression (final staining score < 5) and high-level expression (final staining score ≥ 5).

**Measurement of lactate and ATP levels**

Tumor and cell lysates were prepared as described in the western blotting analysis section. Subsequently, the lactate and ATP contents in the lysate were measured using the Lactate-Glo and ENLITEN ATP assay kits (Promega), respectively. The obtained values were then normalized to the protein concentration.

**Statistical analyses.**

The determination of sample size was predicated on statistical analysis of variance and exploratory experiments. Data are presented as mean ± s.d. or mean ± s.e.m., as described in the respective figure legends. Data were analyzed using the two-sided unpaired *t-test* with Welch’s correction, the Mann–Whitney U-test, or the Fisher’s exact test using GraphPad Prism 9 software (GraphPad Software, Inc., La Jolla, CA, USA), as indicated in each experiment. *p*-values < 0.05 were considered significant. The sample size was based on statistical analysis of variance and exploratory experiments.

**REFERENCES**

1 Kanamori A, Matsubara D, Saitoh Y, Fukui Y, Gotoh N, Kaneko S *et al*. Mint3 depletion restricts tumor malignancy of pancreatic cancer cells by decreasing SKP2 expression via HIF-1. *Oncogene* 2020; 39: 6218-6230.

2 Yoshino S, Matsui Y, Fukui Y, Seki M, Yamaguchi K, Kanamori A *et al*. EXOSC9 depletion attenuates P-body formation, stress resistance, and tumorigenicity of cancer cells. *Sci Rep* 2020; 10: 9275.

3 Yoshino S, Hara T, Nakaoka HJ, Kanamori A, Murakami Y, Seiki M *et al*. The ERK signaling target RNF126 regulates anoikis resistance in cancer cells by changing the mitochondrial metabolic flux. *Cell Discov* 2016; 2: 16019.

4 Hara T, Nakaoka HJ, Hayashi T, Mimura K, Hoshino D, Inoue M *et al*. Control of metastatic niche formation by targeting APBA3/Mint3 in inflammatory monocytes. *Proc Natl Acad Sci U S A* 2017; 114: E4416-E4424.

5 Ikeda J, Ohe C, Tanaka N, Yoshida T, Saito R, Atsumi N *et al*. Hypoxia inducible factor‐1 activator munc‐18‐interacting protein 3 promotes tumour progression in urothelial carcinoma. *Clinical and Translational Discovery* 2023; 3: e158.

6 Conway T, Wazny J, Bromage A, Tymms M, Sooraj D, Williams ED *et al*. Xenome--a tool for classifying reads from xenograft samples. *Bioinformatics* 2012; 28: i172-178.

7 Dobin A, Davis CA, Schlesinger F, Drenkow J, Zaleski C, Jha S *et al*. STAR: ultrafast universal RNA-seq aligner. *Bioinformatics* 2013; 29: 15-21.

8 Li B, Dewey CN. RSEM: accurate transcript quantification from RNA-Seq data with or without a reference genome. *BMC Bioinformatics* 2011; 12: 323.

9 Love MI, Huber W, Anders S. Moderated estimation of fold change and dispersion for RNA-seq data with DESeq2. *Genome Biol* 2014; 15: 550.

10 Langmead B, Salzberg SL. Fast gapped-read alignment with Bowtie 2. *Nat Methods* 2012; 9: 357-359.

11 Zhang Y, Liu T, Meyer CA, Eeckhoute J, Johnson DS, Bernstein BE *et al*. Model-based analysis of ChIP-Seq (MACS). *Genome Biol* 2008; 9: R137.

12 Heinz S, Benner C, Spann N, Bertolino E, Lin YC, Laslo P *et al*. Simple combinations of lineage-determining transcription factors prime cis-regulatory elements required for macrophage and B cell identities. *Mol Cell* 2010; 38: 576-589.
