# Supplementary material for: Mint3-depletion-induced energy stress sensitizes triple-negative breast cancer to chemotherapy via HSF1 inactivation
Source: Cell Death Dis. 2023 Dec 11;14(12):815. doi: 10.1038/s41419-023-06352-4 (PMC10713533; doi:10.1038/s41419-023-06352-4)
Supplement: Supplementary file 2 — Supplementary Fig. S1 [file 41419_2023_6352_MOESM2_ESM.pdf]

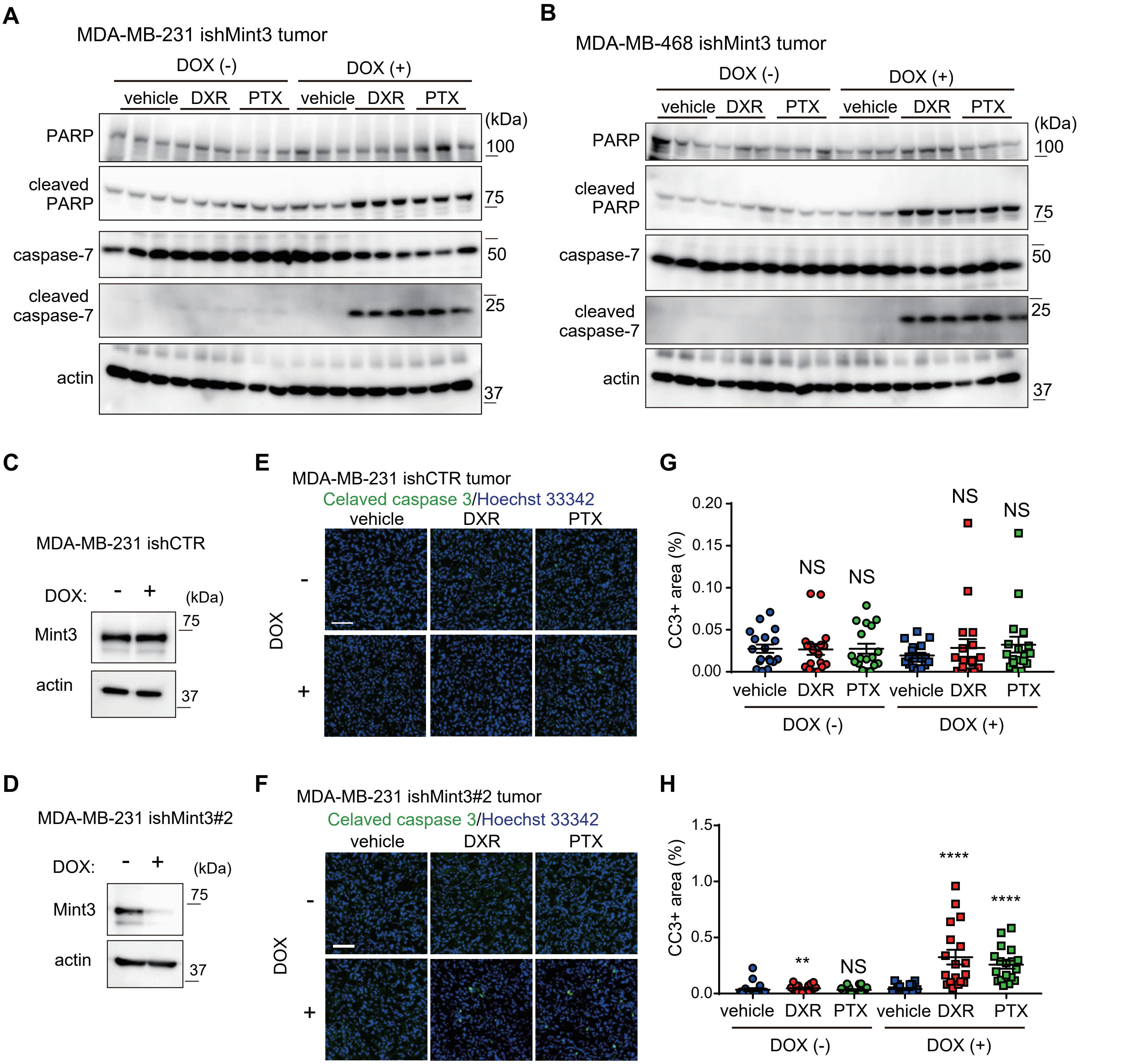

Supplementary Fig. S1. Mint3 depletion sensitizes chemotherapy *in vivo* in MDA-MB-231 cells.
A, **B** Immunoblotting of full-length and cleaved PARP and caspase-7 in tumors from MDA-MB-231 ishMint3#1 (**A**) and MDA-MB-468 ishMint3#1 (**B**) cells following four-day doxycycline (DOX) administration and one-shot chemotherapy with doxorubicin (DXR; 2 mg/kg b.w.) and paclitaxel (PTX; 20 mg/kg b.w.) 24 h before sacrifice, as illustrated in Fig. 2A.
C, **D** Expression of Mint3 in MDA-MB-231 cells expressing control shRNA (ishCTR) or shRNA against Mint3 with a different sequence from ishMint3 (ishMint3#2) treated with DOX (1 μg/ml) for three days.
E–**H** Immunostaining for cleaved caspase-3 in tumors from MDA-MB-231 ishCTR (**E**, **G**) and ishMint3#2 (**F**, **H**) cells following four-day doxycycline (DOX) administration and one-shot chemotherapy with DXR and PTX 24 h before sacrifice, as illustrated in Fig. 2A. (**E**, **F**) Representative images are shown. (**G**, **H**) Cleaved caspase-3-positive areas were counted in the tumor sections. n = 18 from six tumors per group. Data are presented as the mean ± SEM and were analyzed using the Mann–Whitney U-test. $^{**}p < 0.01$, $^{****}p < 0.0001$. NS, not significant.
