# Supplementary material for: Mint3-depletion-induced energy stress sensitizes triple-negative breast cancer to chemotherapy via HSF1 inactivation
Source: Cell Death Dis. 2023 Dec 11;14(12):815. doi: 10.1038/s41419-023-06352-4 (PMC10713533; doi:10.1038/s41419-023-06352-4)
Supplement: Supplementary file 3 — Supplementary Fig. S2 [file 41419_2023_6352_MOESM3_ESM.pdf]

A

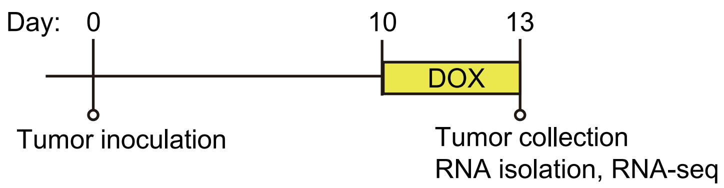

B

Rank	Gene	log2 FC	adjusted p value
1	RSC1A1	-1.183149837	0.007955842
2	HCLS1	-0.898211842	0.029852346
3	ID2	-0.871320171	0.027769149
4	HSPA1A	-0.829165386	0.002898218
5	TNFSF18	-0.784984533	0.00189841
6	DGKI	-0.703953292	0.033411853
7	FSBP	-0.678040422	0.031616898
8	MAP3K7CL	-0.607979805	5.64E-05

Underrepresented genes in Mint3-depleted MDA-MB-231 tumors

C

Rank	Gene	log2 FC	adjusted p value
1	S100A4	0.633325175	5.38E-18
2	IFI27	0.672891278	0.000140619
3	SYT12	0.678921567	0.029852346
4	ANXA8L1	0.72905626	0.043515559
5	KRT17	1.004037298	1.70E-06
6	KRCC1	1.078352498	0.003749548
7	GATD3A	1.159826405	0.037626749
8	PI16	1.221938956	0.043786295
9	KRT14	1.390402905	0.008722136

Overerepresented genes in Mint3-depleted MDA-MB-231 tumors

D

MDA-MB-231 ishCTR tumor

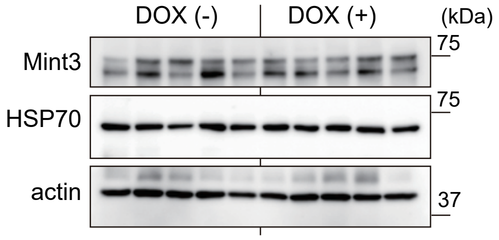

E

MDA-MB-231 ishMint3#2 tumor

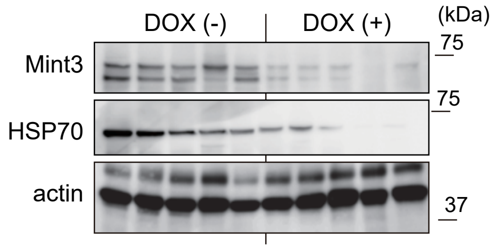

F

MDA-MB-231 tumor

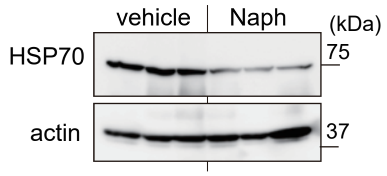

Supplementary Fig. S2. Mint3 promotes HSP70 expression in tumors from MDA-MB-231 cells.
A Schematic illustration of the RNA-seq analysis of MDA-MB-231 ishMint3 tumors.
B List of underrepresented genes in Mint3-depleted MDA-MB-231 tumors.
C List of overrepresented genes in Mint3-depleted MDA-MB-231 tumors.
D, **E** Protein levels of Mint3 and HSP70 in tumors from MDA-MB-231 ishCTR (**D**) and ishMint3#2 cells (**E**) administered with or without doxycycline (DOX) for 3 days. n = 5 per group.
F Protein levels of HSP70 in tumors from MDA-MB-231 cells administered with or without naphthofluorescein (Naph) for three days. n = 3 per group.
