# Supplementary material for: Mint3-depletion-induced energy stress sensitizes triple-negative breast cancer to chemotherapy via HSF1 inactivation
Source: Cell Death Dis. 2023 Dec 11;14(12):815. doi: 10.1038/s41419-023-06352-4 (PMC10713533; doi:10.1038/s41419-023-06352-4)
Supplement: Supplementary file 4 — Supplementary Fig. S3 [file 41419_2023_6352_MOESM4_ESM.pdf]

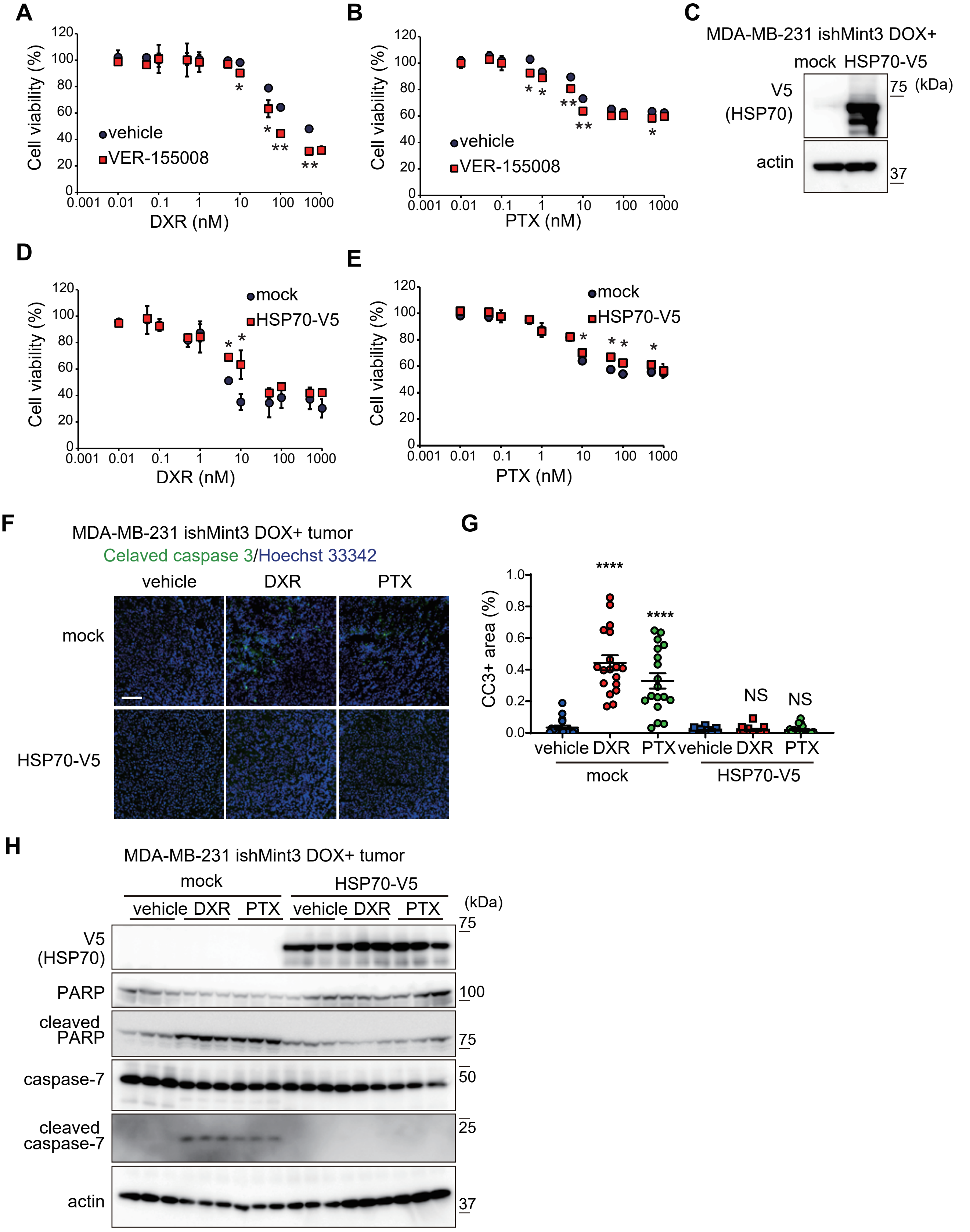

Supplementary Fig. S3. HSP70 contributes to chemoresistance in MDA-MB-231 cells both i*n vitro* and *in vivo*.

A, **B** Cytotoxicity assay of MDA-MB-231 ishMint3 cells in the presence of doxorubicin (DXR) or paclitaxel (PTX) with or without VER-155008 (10 μM) for 24 h at the indicated concentration.

C Immunoblotting of V5-tagged HSP70 in MDA-MB-231 ishMint3 cells transfected with mock or HSP70-V5 expressing lentiviral vectors in the presence of doxycycline (DOX).

D, **E** Cytotoxicity assay of MDA-MB-231 ishMint3 mock and HSP70-V5 cells in the presence of DXR or PTX with DOX for 24 h at the indicated concentrations.

F, **G** Immunostaining for cleaved caspase-3 in tumors derived from MDA-MB-231 ishMint3 mock and HSP70-V5 cells following four-day DOX administration, accompanied by a single-shot chemotherapeutic application of DXR and PTX 24 h before sacrifice. (**F**) representative photos. (**G**) Quantification of cleaved caspase-3-positive regions in the tumor sections, with n = 18, obtained from six tumors per group. All data are presented as the mean ± SEM and were analyzed using the Mann–Whitney U-test. ****$p < 0.0001$. NS: not significant.

For Panels **A**, **B**, **D**, and **E**, data are presented as the mean ± SD (n = 3) and analyzed using the two-sided unpaired t-test with Welch' s correction. *$p < 0.05$, **$p < 0.01$.
