# Supplementary material for: Mint3-depletion-induced energy stress sensitizes triple-negative breast cancer to chemotherapy via HSF1 inactivation
Source: Cell Death Dis. 2023 Dec 11;14(12):815. doi: 10.1038/s41419-023-06352-4 (PMC10713533; doi:10.1038/s41419-023-06352-4)
Supplement: Supplementary file 5 — Supplementary Fig. S4 [file 41419_2023_6352_MOESM5_ESM.pdf]

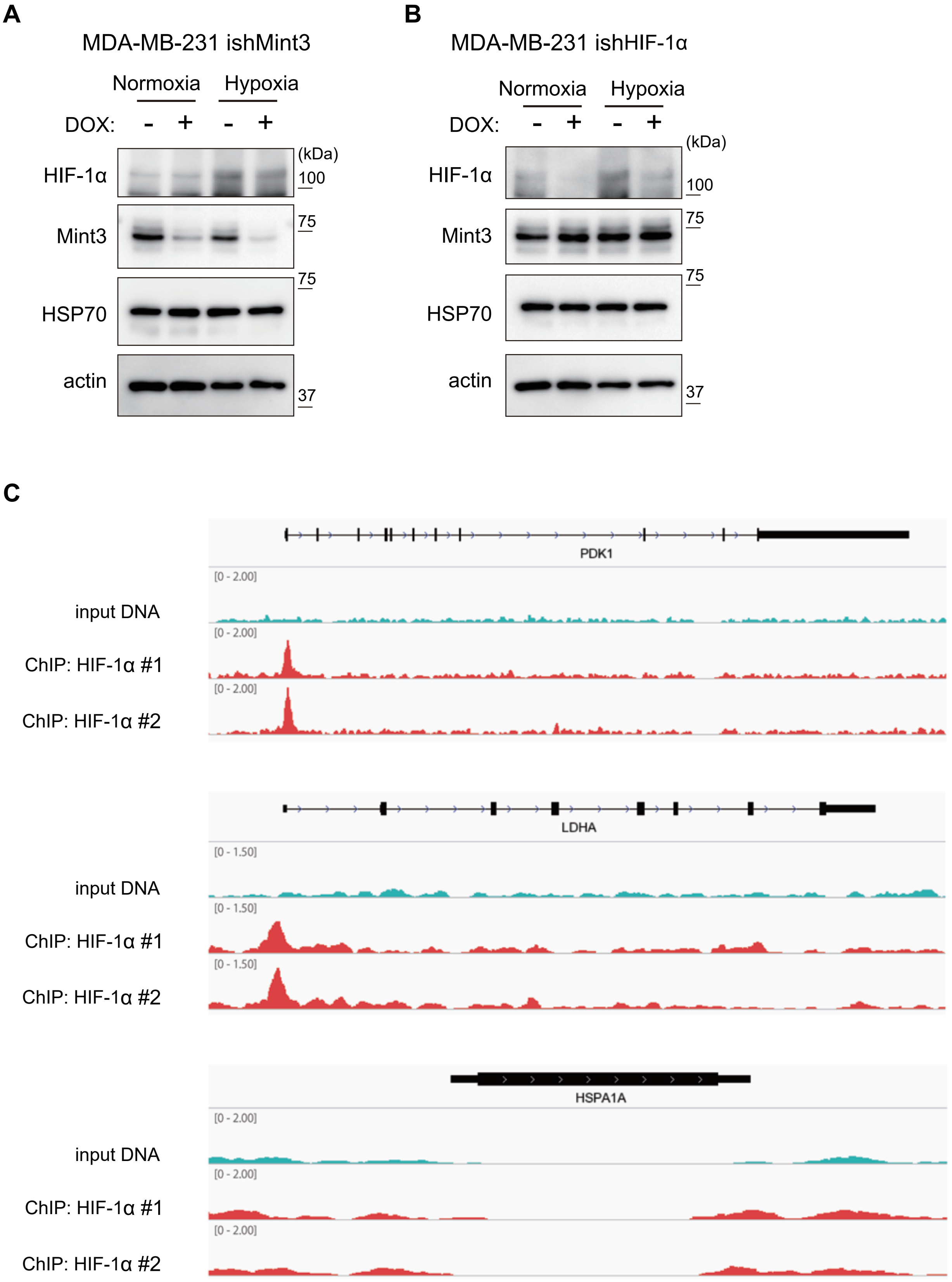

Supplementary Fig. S4. Hypoxia alone does not induce HSP70 in TNBC cells *in vitro*.
A, **B** Protein levels of HIF-1α, Mint3, and HSP70 in MDA-MB-231 ishMint3 (**A**) and ishHIF-1α cells (**B**) with or without doxycycline and cultured under normoxic or hypoxic conditions for 24 h.
C ChIP-seq analysis of known HIF-1 target genes and *HSPA1A* in tumors from MDA-MB-231 cells using anti-HIF-1α antibodies. Two independent precipitants of anti-HIF-1α antibodies were analyzed. Note that there is no apparent peak of anti-HIF-1α antibodies in the locus of the *HSPA1A* gene.
