# Supplementary material for: Mint3-depletion-induced energy stress sensitizes triple-negative breast cancer to chemotherapy via HSF1 inactivation
Source: Cell Death Dis. 2023 Dec 11;14(12):815. doi: 10.1038/s41419-023-06352-4 (PMC10713533; doi:10.1038/s41419-023-06352-4)
Supplement: Supplementary file 6 — Supplementary Fig. S5 [file 41419_2023_6352_MOESM6_ESM.pdf]

MDA-MB-231 ishHIF-1α tumor

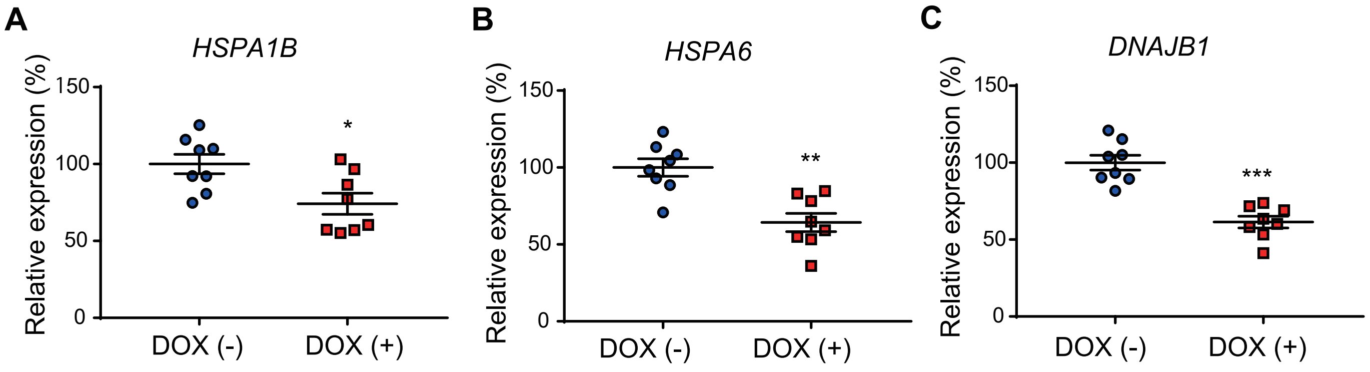

Supplementary Fig. S5. HIF-1 promotes the expression of HSF-1 target genes in TNBC tumors.
A–**C** mRNA levels of HSF1 target genes (*HSPA1B* (**A**), *HSPA6* (**B**), and *DNAJB1* (**C**)) in tumors of MDA-MB-231 ishHIF-1α cells treated with or without doxycycline (DOX). Data are presented as the mean ± SEM and were analyzed using the Mann–Whitney U-test. *$p < 0.05$, **$p < 0.01$, ***$p < 0.001$.
