# Supplementary material for: Mint3-depletion-induced energy stress sensitizes triple-negative breast cancer to chemotherapy via HSF1 inactivation
Source: Cell Death Dis. 2023 Dec 11;14(12):815. doi: 10.1038/s41419-023-06352-4 (PMC10713533; doi:10.1038/s41419-023-06352-4)
Supplement: Supplementary file 7 — Supplementary Fig. S6 [file 41419_2023_6352_MOESM7_ESM.pdf]

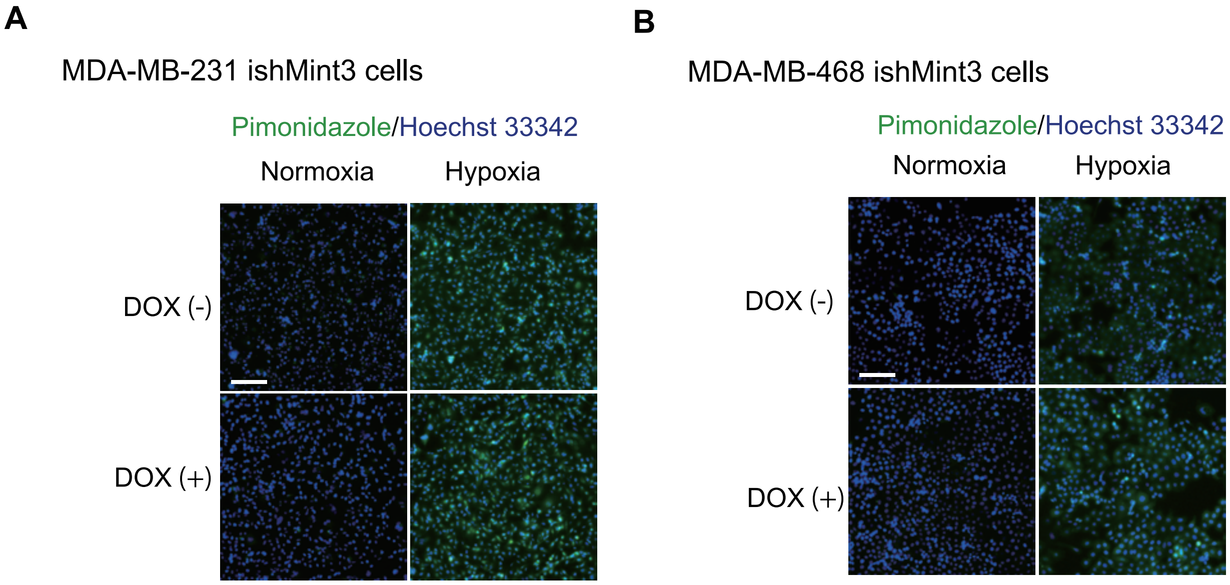

Supplementary Fig. S6. Mint3 depletion did not induce hypoxia *in vitro* in MDA-MB-231 and MDA-MB-468 cells. Immunostaining of pimonidazole (green) in MDA-MB-231 ishMint3#1 (**A**) and MDA-MB-468 ishMint3#1 (**B**) cells, with or without doxycycline (DOX), cultured under normoxic or hypoxic conditions for 3 h in the presence of pimonidazole (100 μM). Nuclei were counterstained with Hoechst 33342 (blue). Scale bar = 100 μm.
