# Supplementary material for: Mint3-depletion-induced energy stress sensitizes triple-negative breast cancer to chemotherapy via HSF1 inactivation
Source: Cell Death Dis. 2023 Dec 11;14(12):815. doi: 10.1038/s41419-023-06352-4 (PMC10713533; doi:10.1038/s41419-023-06352-4)
Supplement: Supplementary file 8 — Supplementary Fig. S7 [file 41419_2023_6352_MOESM8_ESM.pdf]

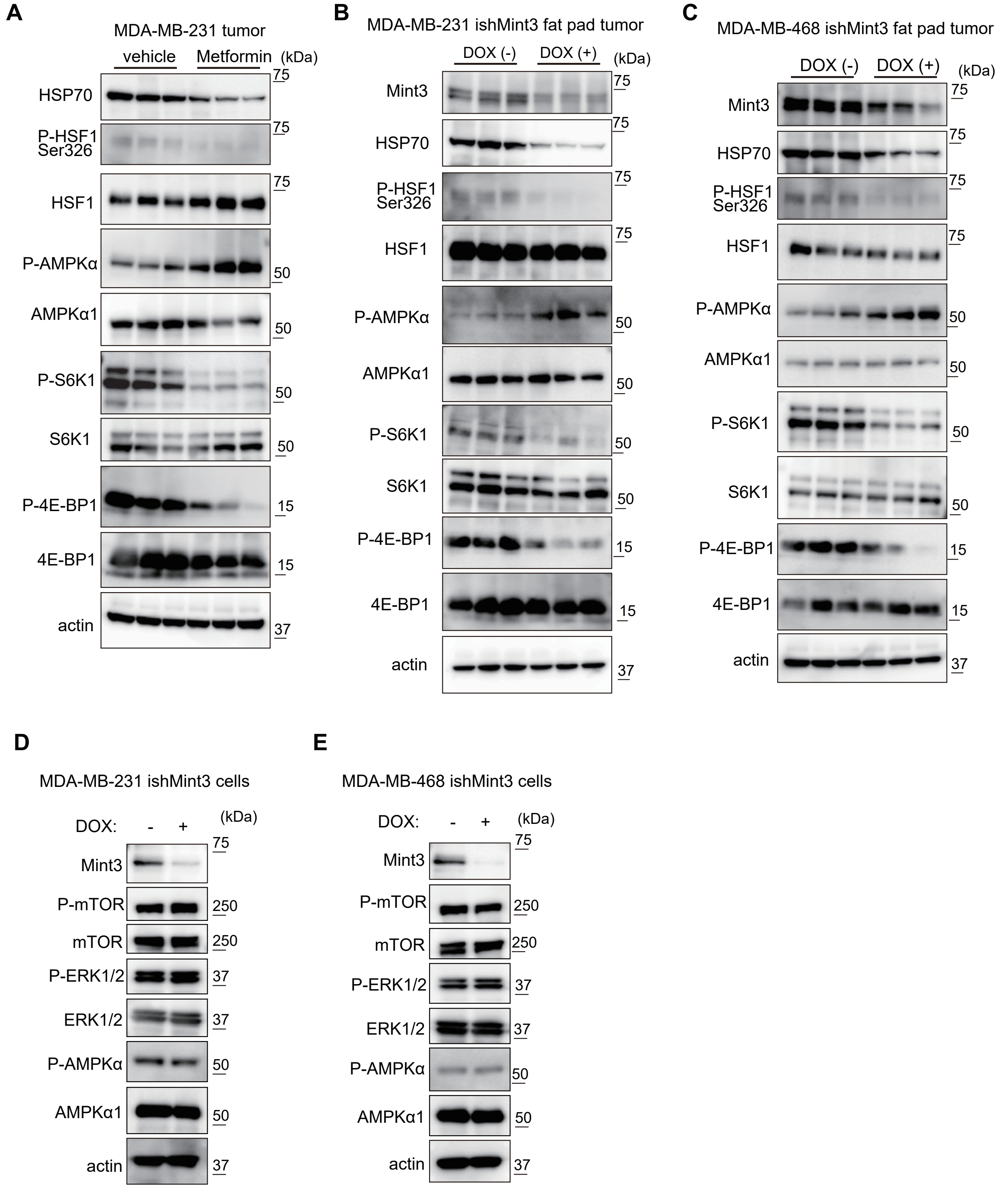

Supplementary Fig. S7. Mint3 depletion activates AMPK1 in TNBC in an in vivo-specific manner.

A Immunoblotting of HSP-70, AMPK, and mTOR signaling-related proteins in MDA-MB-231 tumors administered with or without metformin for three days. n = 3 per group.

B, **C** Immunoblotting of HSF-1 and its upstream signaling-related proteins in MDA-MB-231 ishMint3 (**B**) and MDA-MB-468 ishMint3 (**C**) tumors grown in the mammary fat pad, treated with or without DOX for three days. n = 3 per group.

D, **E** Immunoblotting of total and phosphorylated mTOR, AMPK1, and ERK1/2 in MDA-MB-231 ishMint3 (**D**) and MDA-MB-468 ishMint3 (**E**) cells, treated with or without DOX for three days.
