# Supplementary material for: Mint3-depletion-induced energy stress sensitizes triple-negative breast cancer to chemotherapy via HSF1 inactivation
Source: Cell Death Dis. 2023 Dec 11;14(12):815. doi: 10.1038/s41419-023-06352-4 (PMC10713533; doi:10.1038/s41419-023-06352-4)
Supplement: Supplementary file 9 — Supplementary Fig. S8 [file 41419_2023_6352_MOESM9_ESM.pdf]

MDA-MB-231 cells

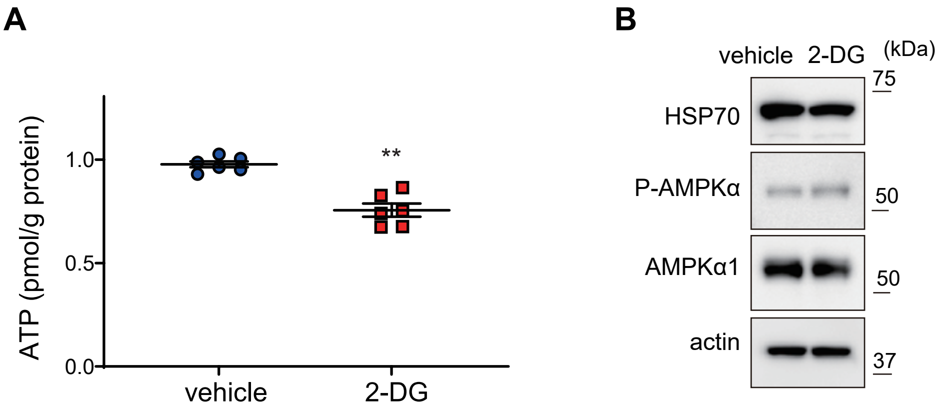

Supplementary Fig. S8. 2-DG partially decreases ATP levels in MDA-MB-231 cells
A, B MDA-MB-231 cells were treated with or without 2-DG (1 mM) for 6 h, and ATP levels (**A**) and protein expression (**B**) were analyzed. (**A**) ATP levels in MDA-MB-231 cells. n = 6 per group. Data are represented as the mean ± SEM and analyzed using the Mann–Whitney U-test. **p < 0.01, ***p < 0.001, ****p < 0.0001. NS: not significant. (**B**) protein expression of HSP70, P-AMPKα, and AMPKα1 in MDA-MB-231 cells.
