# Supplementary material for: Mint3-depletion-induced energy stress sensitizes triple-negative breast cancer to chemotherapy via HSF1 inactivation
Source: Cell Death Dis. 2023 Dec 11;14(12):815. doi: 10.1038/s41419-023-06352-4 (PMC10713533; doi:10.1038/s41419-023-06352-4)
Supplement: Supplementary file 11 — Supplementary Table S2 [file 41419_2023_6352_MOESM11_ESM.docx]

| *ACTB* | Forward (f) | 5′- TTCTACAATGAGCTGCGTGTG -3′ |
| --- | --- | --- |
|  | Reverse (r) | 5′- GGGGTGTTGAAGGTCTCAAA -3′ |
| *APBA3* | f | AAGAGGATCAAGGTCTTGAC |
|  | r | GTGTAGGAGATGGTATGCAG |
| *HSPA1A* | f | TAACCCCATCATCAGCGGAC |
|  | r | AACAGCAATCTTGGAAAGGCCC |
| *HSPA1B* | f | GGTGGATTAGGGGCCTTTGT |
|  | r | ACAGCAGCAAAGTCCTTGAGT |
| *HSPA6* | f | CAGAGGAACGCCACTATCCC |
|  | r | ACTGAGTTCAAAACGCCCCA |
| *DNAJB1* | f | AGGGGGTCATGGGTAAAGACT |
|  | r | GGGCTCCTTGTTCTTGTCCG |
| *HIF1A* | f | ATCCATGTGACCATGAGGAAATG |
|  | r | CTCGGCTAGTTAGGGTACACTT |

**Supplementary Table S2**. **Primer pairs used in quantitative real-time polymerase chain reaction.**
