# Supplementary material for: Mint3-depletion-induced energy stress sensitizes triple-negative breast cancer to chemotherapy via HSF1 inactivation
Source: Cell Death Dis. 2023 Dec 11;14(12):815. doi: 10.1038/s41419-023-06352-4 (PMC10713533; doi:10.1038/s41419-023-06352-4)

Fig. 1

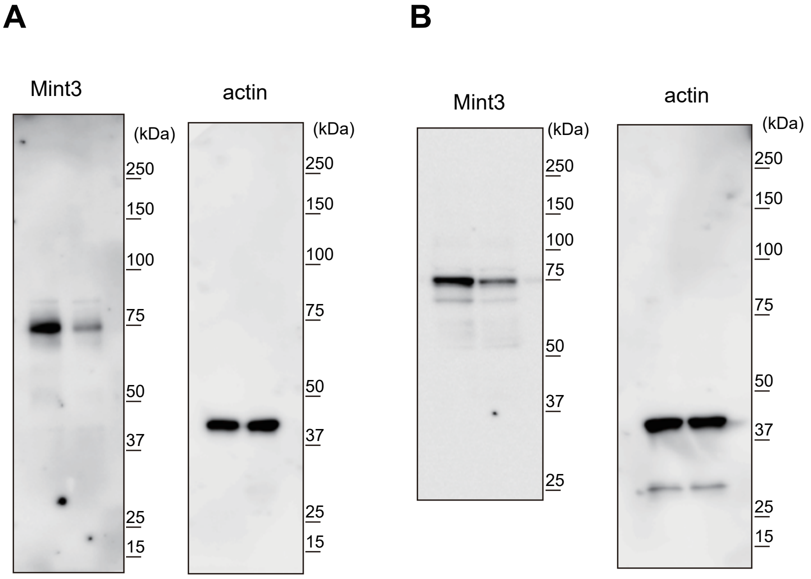
A
B
Mint3
actin
(kDa)
250
150
100
75
50
37
25
15

Fig. 3

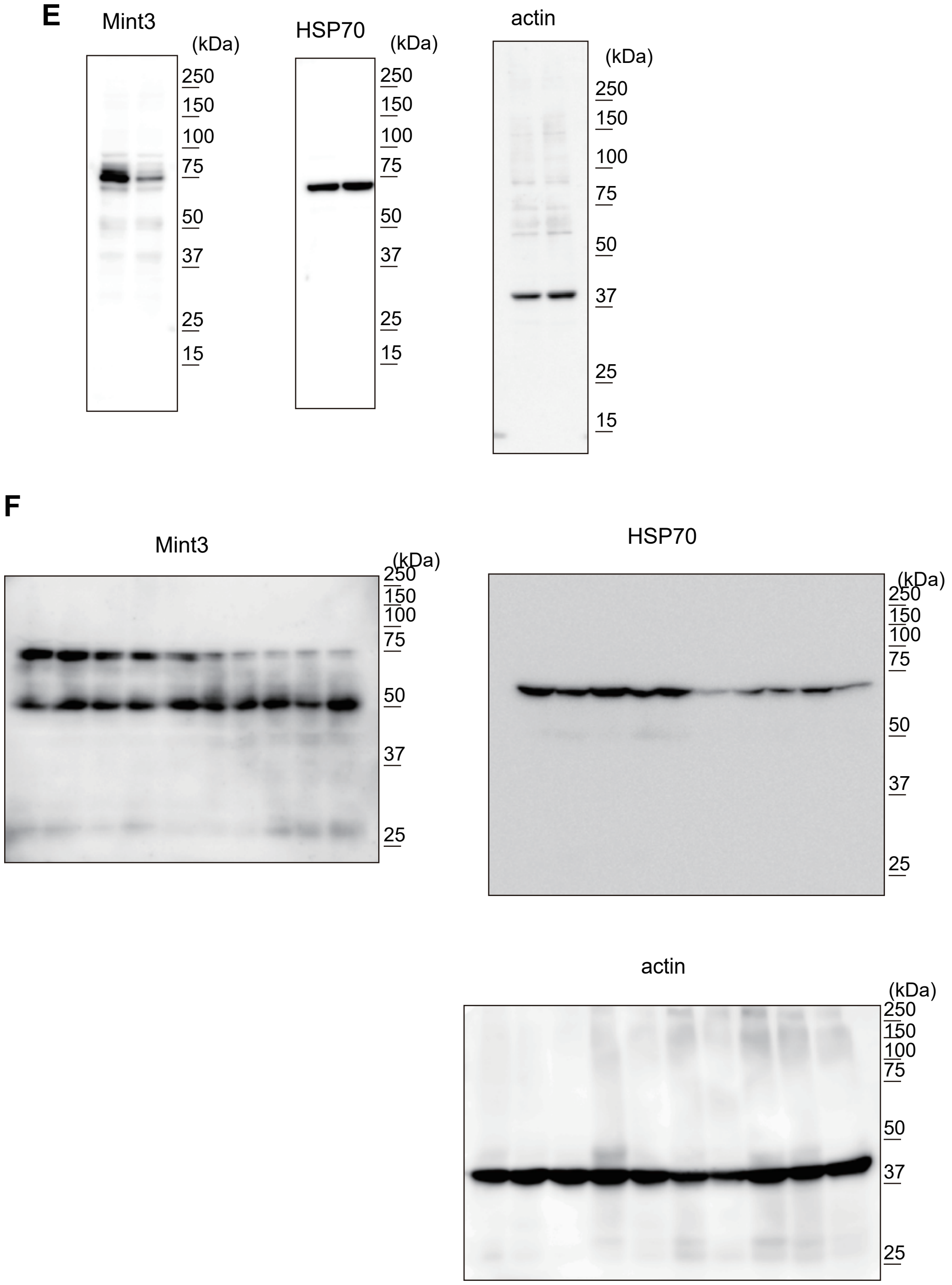
E
F
Mint3
HSP70
actin
(kDa)
250
150
100
75
50
37
25
15

Fig. 3

G

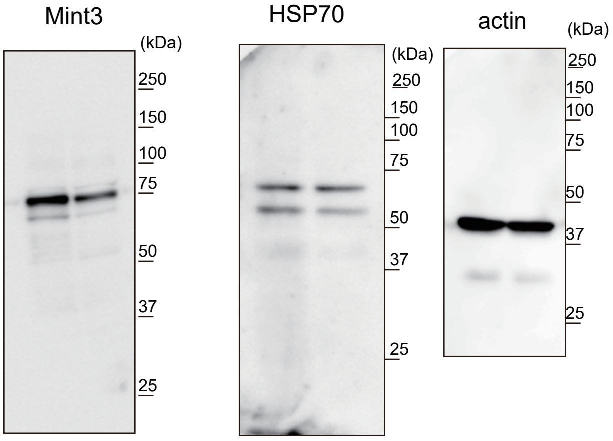
Mint3
(kDa)
250
150
100
75
50
37
25
HSP70
(kDa)
250
150
100
75
50
37
25
actin
(kDa)
250
150
100
75
50
37
25

H

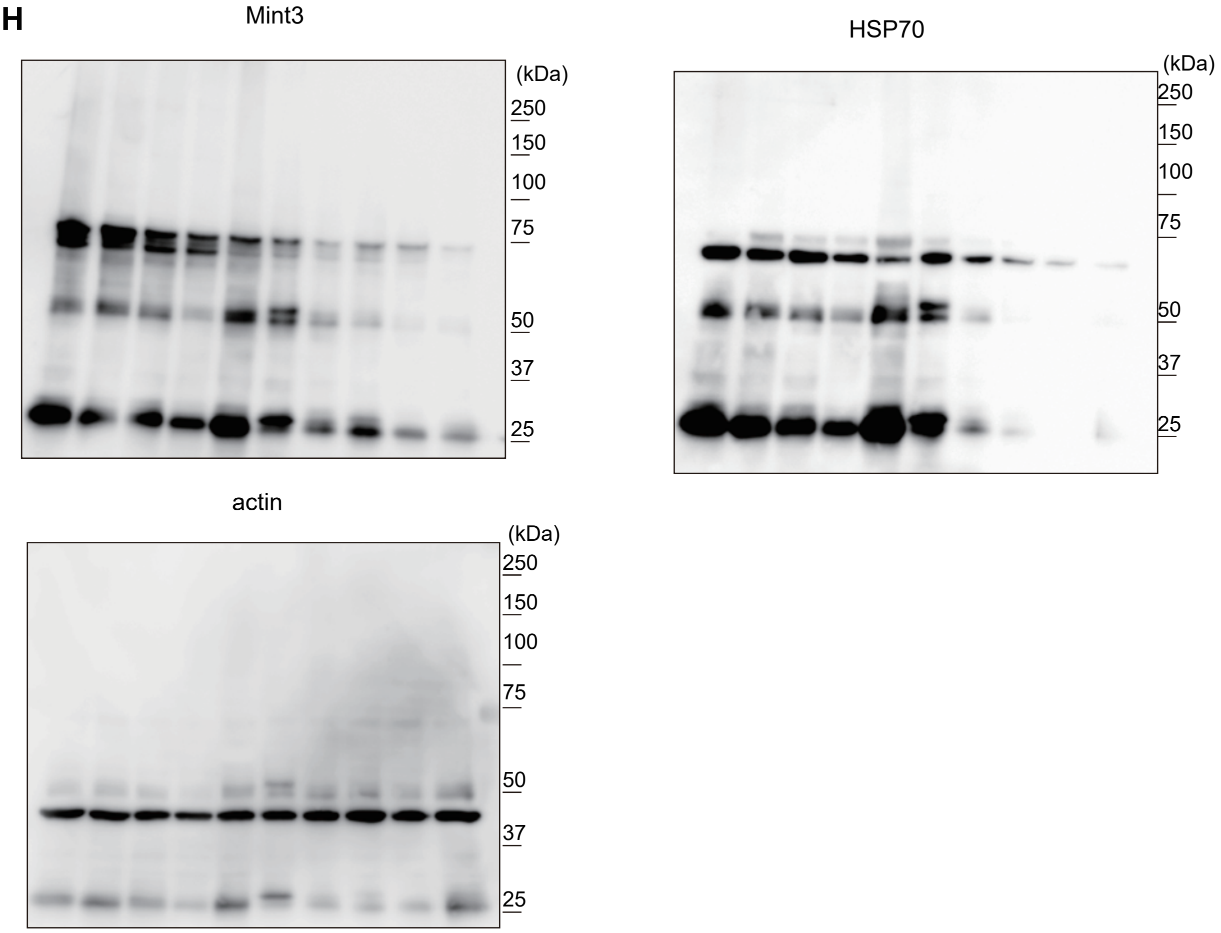
Mint3
(kDa)
250
150
100
75
50
37
25
HSP70
(kDa)
250
150
100
75
50
37
25
actin
(kDa)
250
150
100
75
50
37
25

Fig. 5

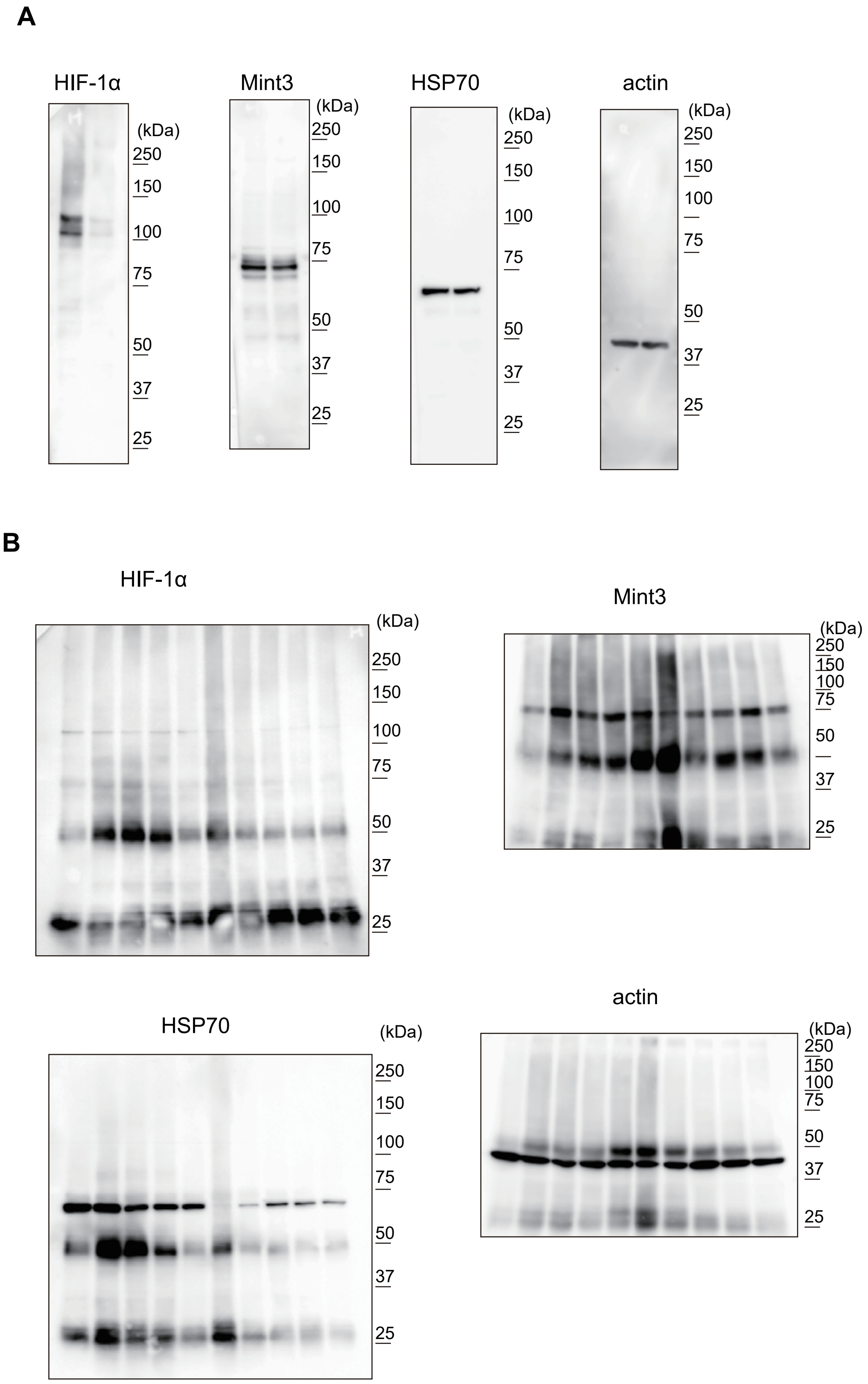
A
HIF-1α
Mint3
HSP70
actin
(kDa)
250
150
100
75
50
37
25
B
HIF-1α
Mint3
HSP70
actin

Fig. 6

G

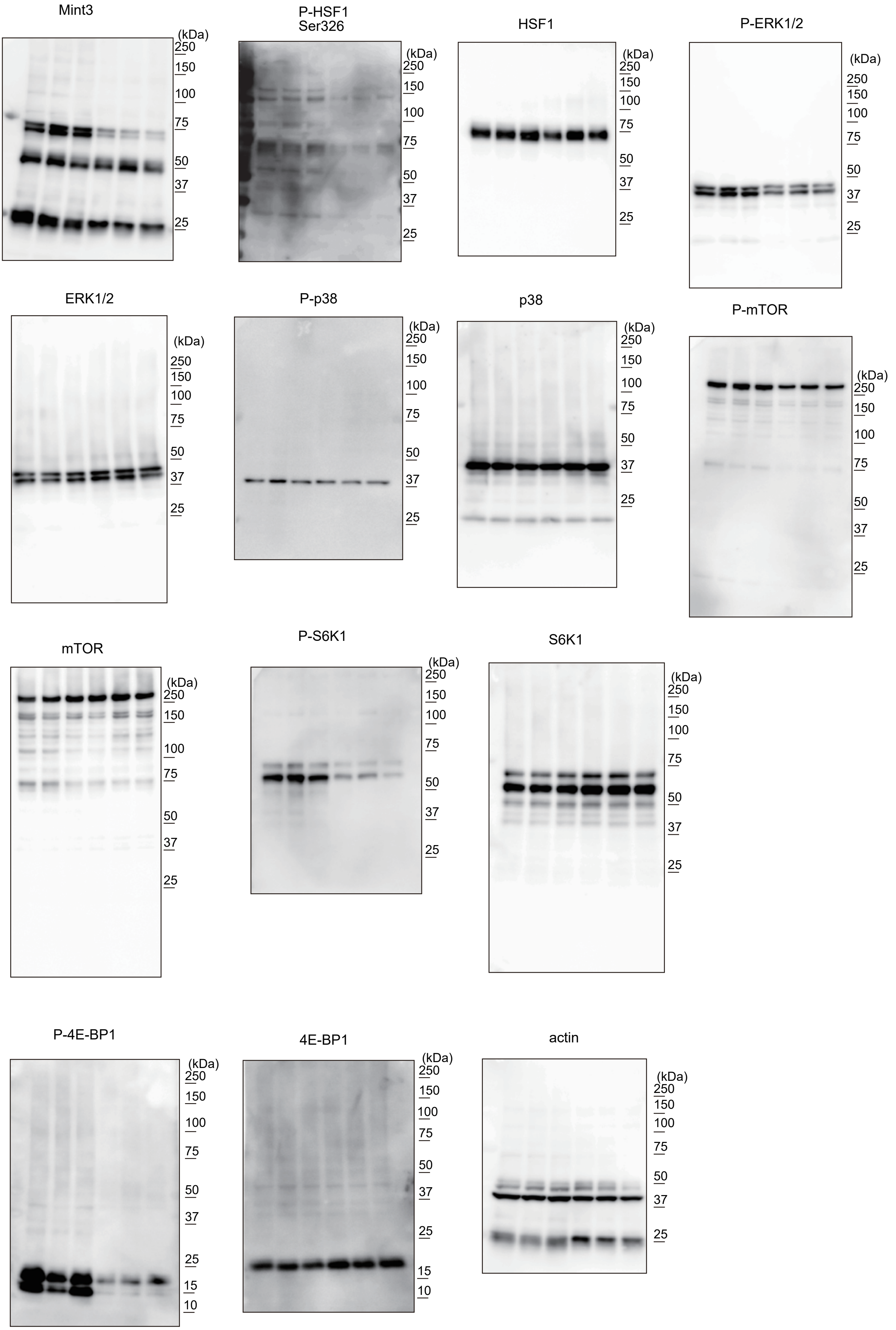
Mint3
(kDa)
250
150
100
75
50
37
25
P-HSF1
Ser326
(kDa)
250
150
100
75
50
37
25
HSF1
(kDa)
250
150
100
75
50
37
25
P-ERK1/2
(kDa)
250
150
100
75
50
37
25
ERK1/2
(kDa)
250
150
100
75
50
37
25
P-p38
(kDa)
250
150
100
75
50
37
25
p38
(kDa)
250
150
100
75
50
37
25
P-mTOR
(kDa)
250
150
100
75
50
37
25
mTOR
(kDa)
250
150
100
75
50
37
25
P-S6K1
(kDa)
250
150
100
75
50
37
25
S6K1
(kDa)
250
150
100
75
50
37
25
P-4E-BP1
(kDa)
250
150
100
75
50
37
25
15
10
4E-BP1
(kDa)
250
150
100
75
50
37
25
15
10
actin
(kDa)
250
150
100
75
50
37
25

Fig. 6

H

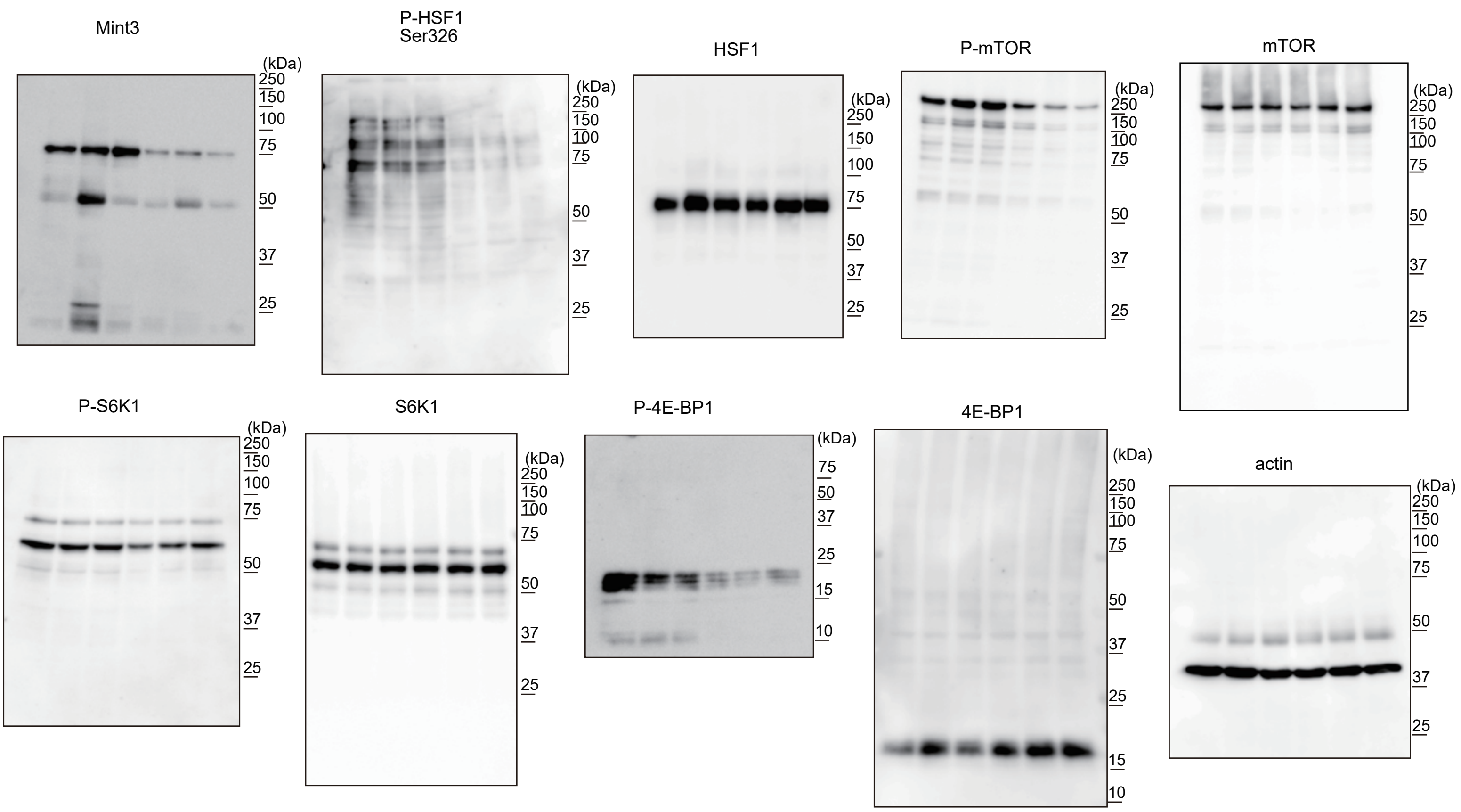

Mint3
P-HSF1
Ser326
HSF1
P-mTOR
mTOR
P-S6K1
S6K1
P-4E-BP1
4E-BP1
actin
(kDa)
250
150
100
75
50
37
25
15
10

I

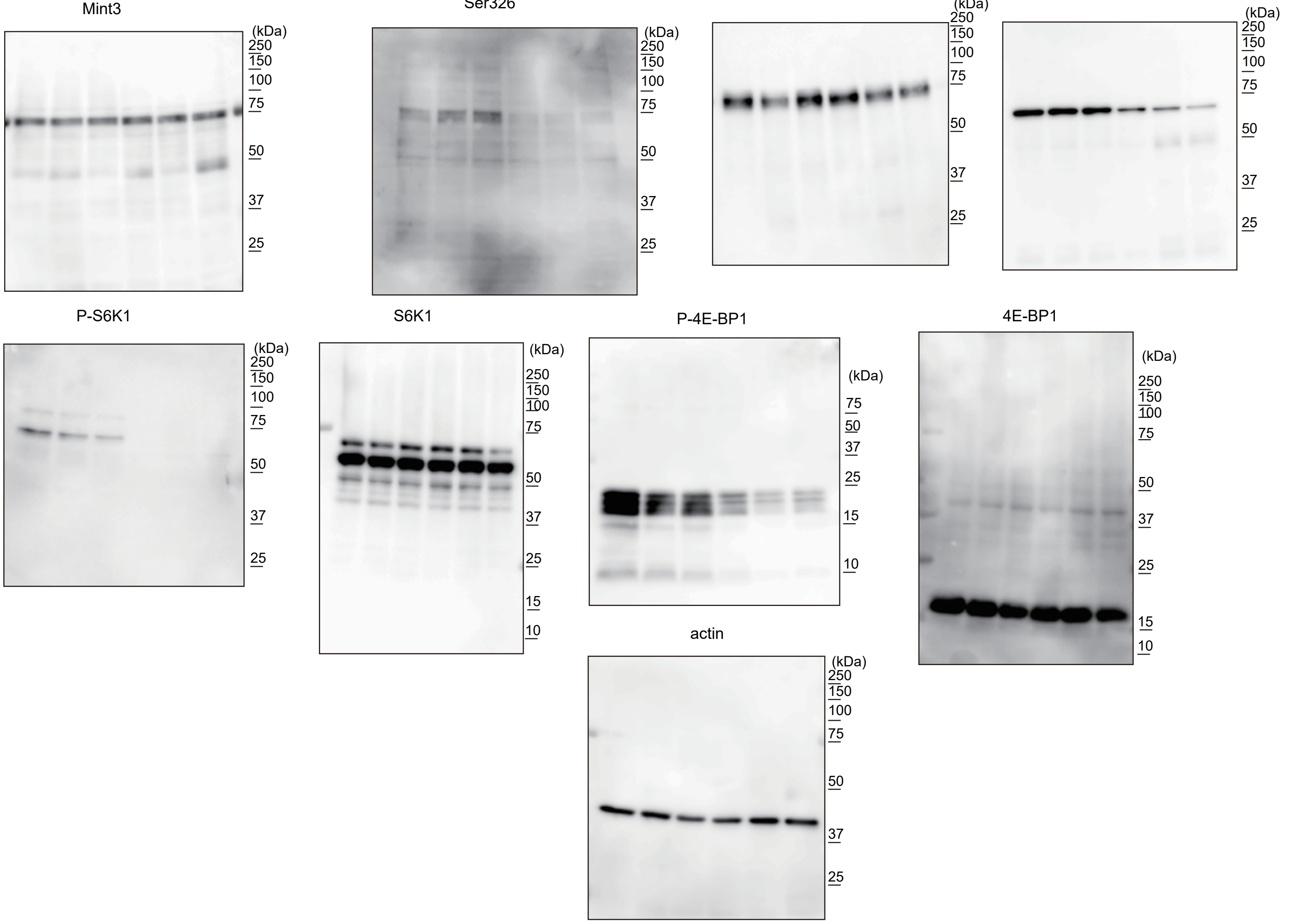

Mint3
P-HSF1
Ser326
HSF1
HSP70
P-S6K1
S6K1
P-4E-BP1
4E-BP1
actin
(kDa)
250
150
100
75
50
37
25
15
10

Fig. 7

G

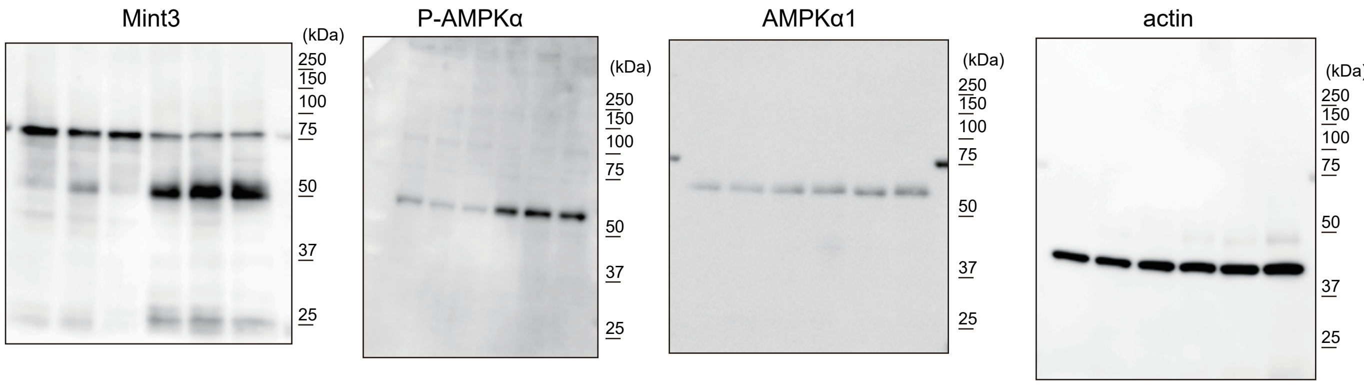
Mint3
P-AMPKα
AMPKα1
actin
(kDa)
250
150
100
75
50
37
25

H

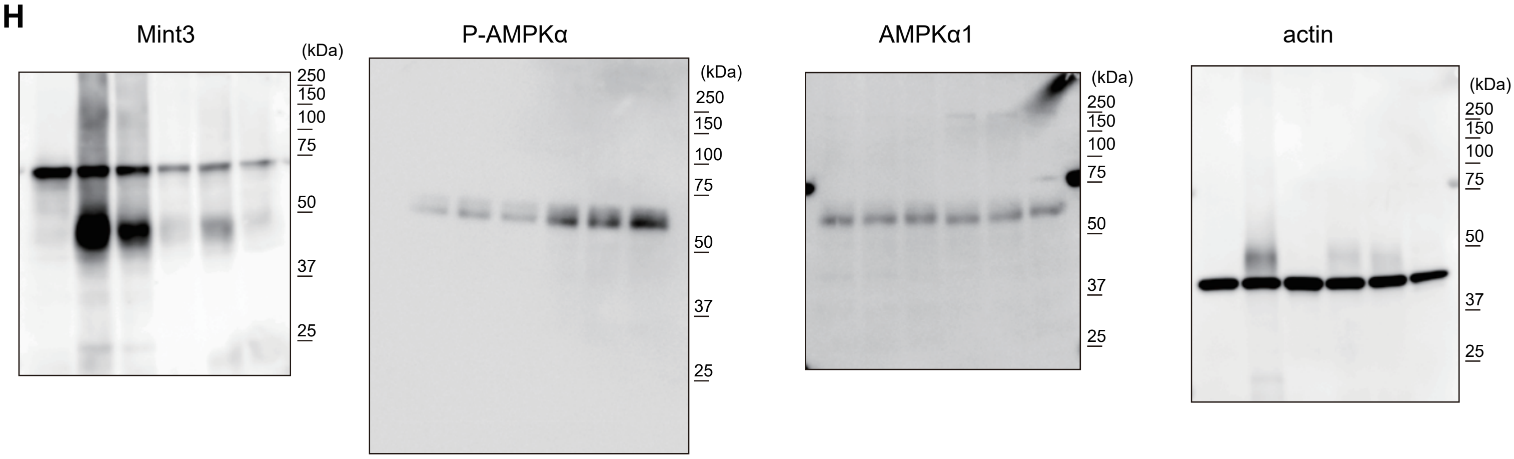
Mint3
P-AMPKα
AMPKα1
actin
(kDa)
250
150
100
75
50
37
25

L

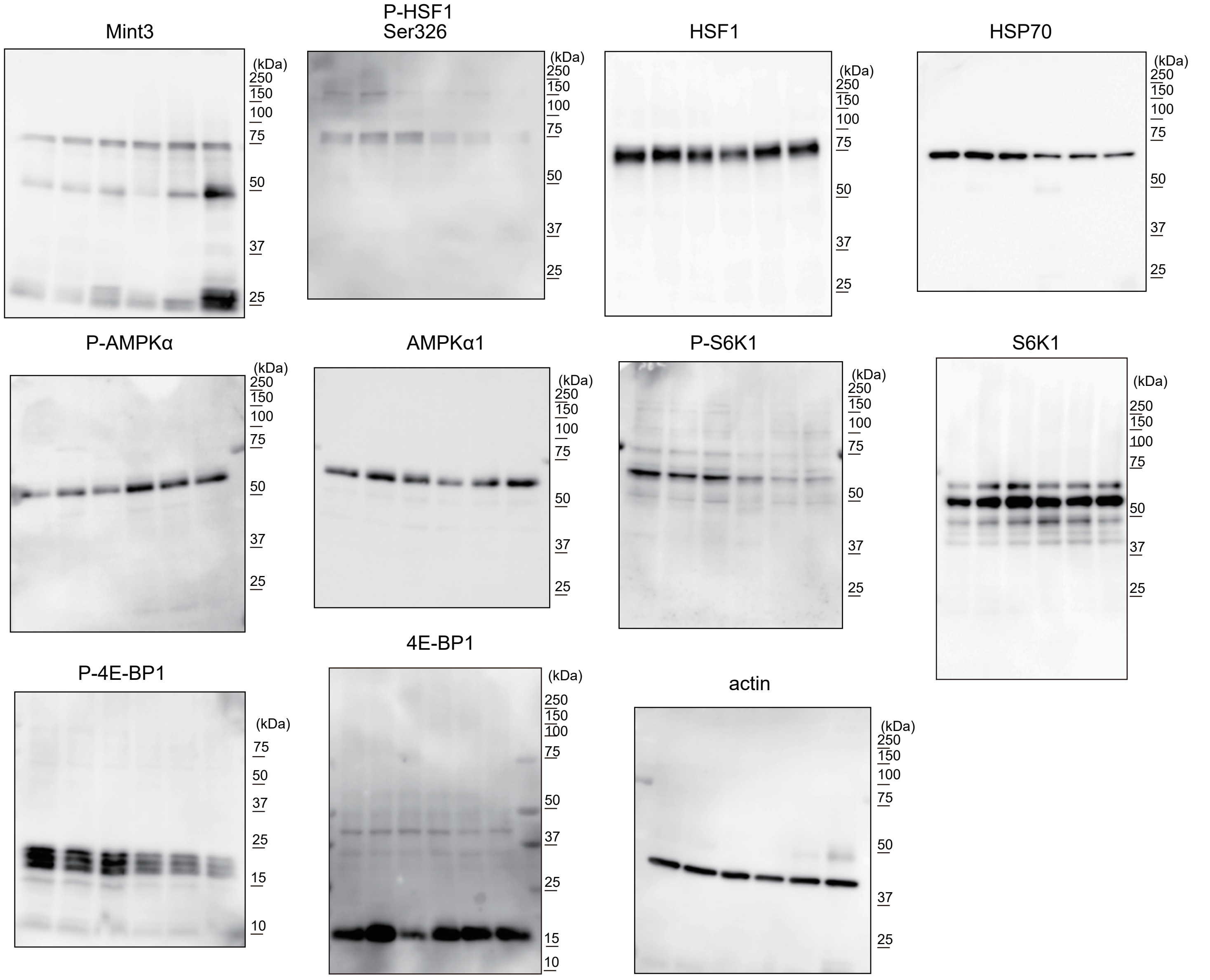
Mint3
P-HSF1
Ser326
HSF1
HSP70
P-AMPKα
AMPKα1
P-S6K1
S6K1
P-4E-BP1
4E-BP1
actin
(kDa)
250
150
100
75
50
37
25
15
10

Supplementary Fig. S1.

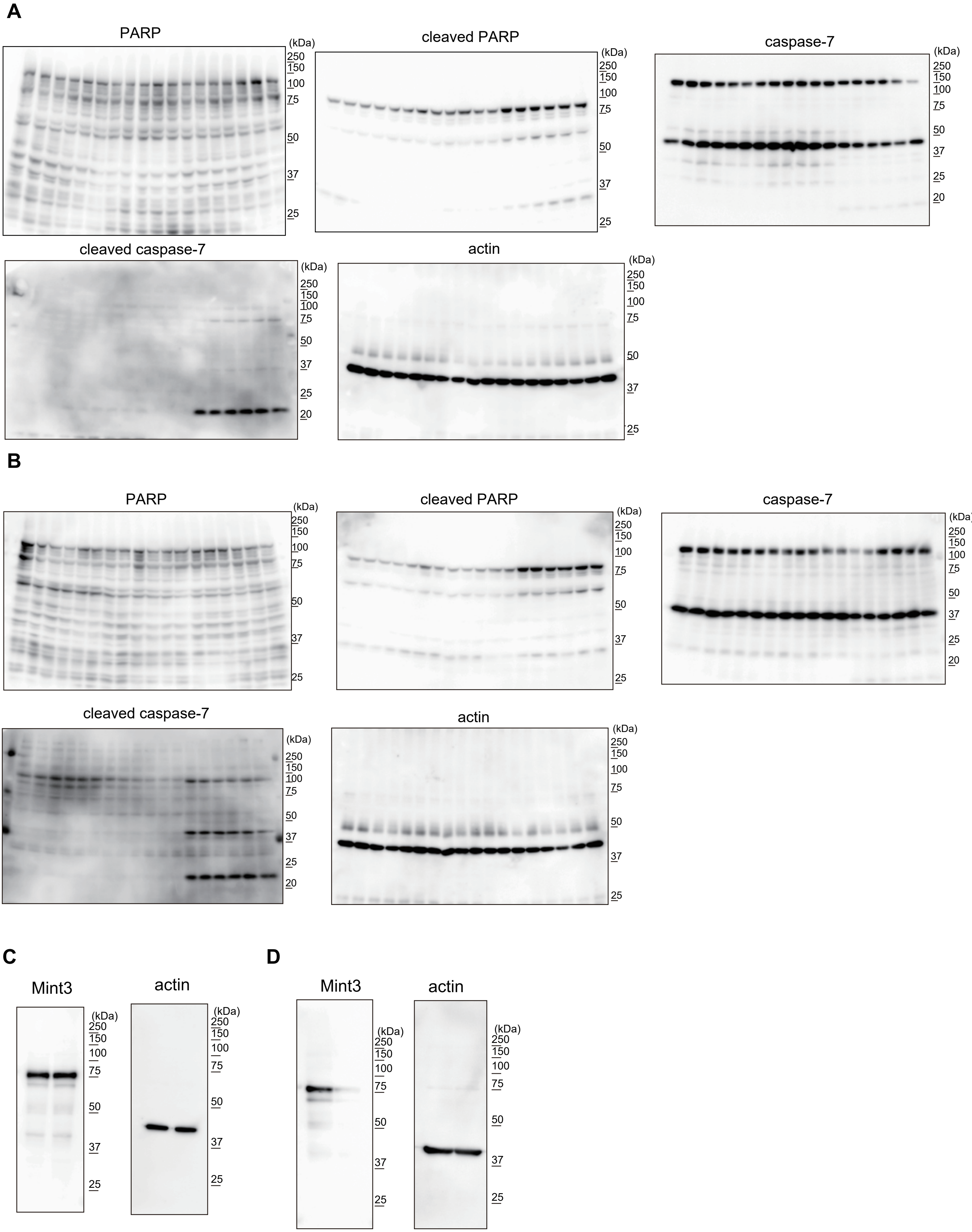

Supplementary Fig. S2.

D

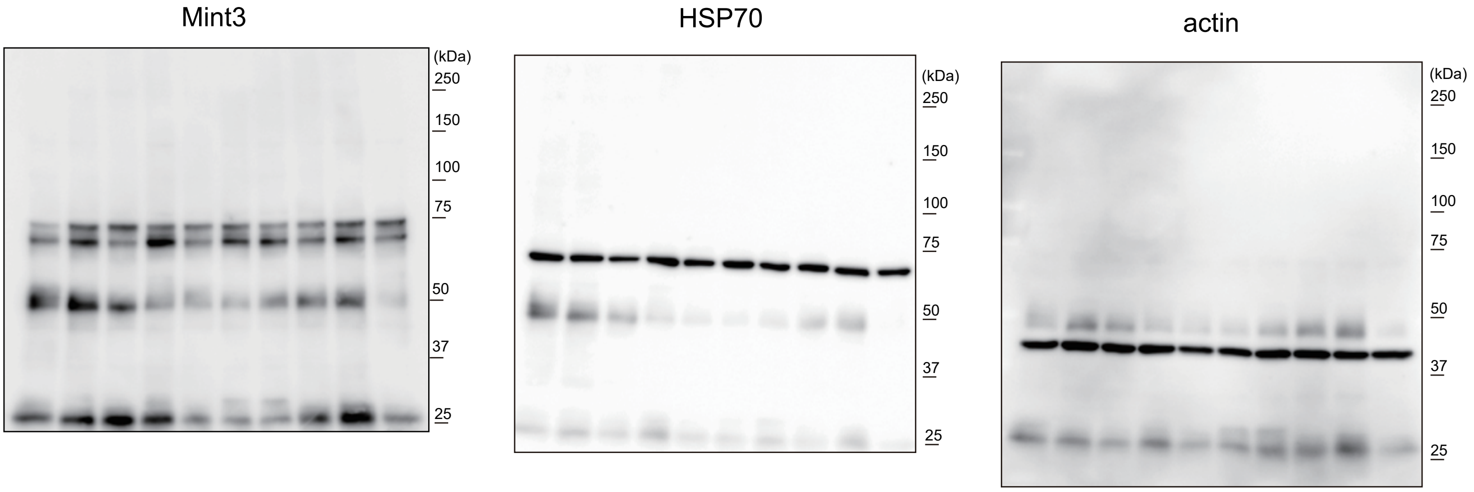

E

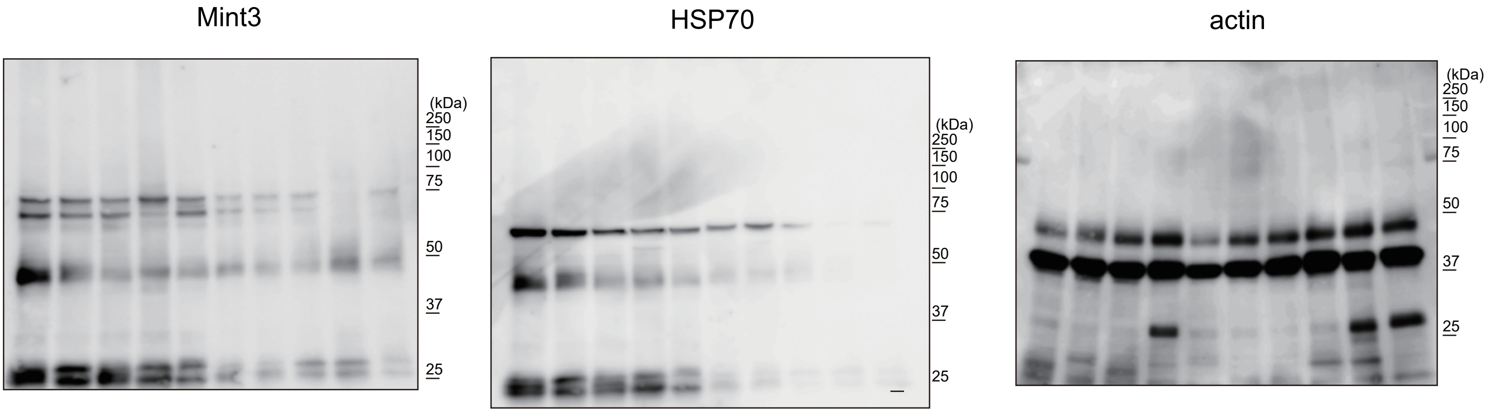

F

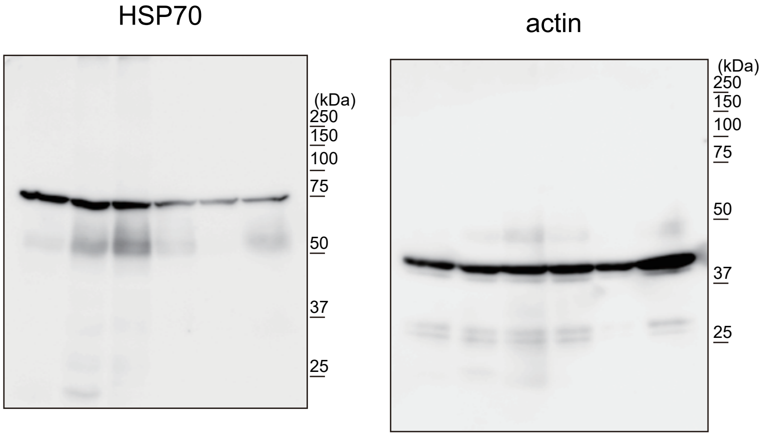

Supplementary Fig. S3

C

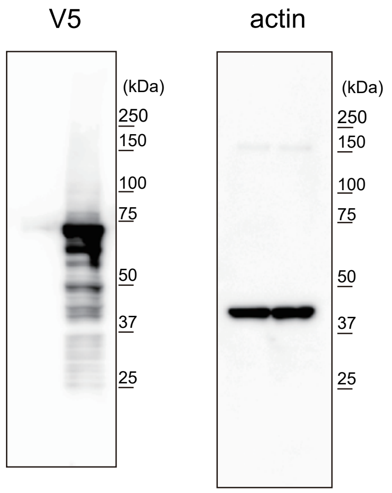

H

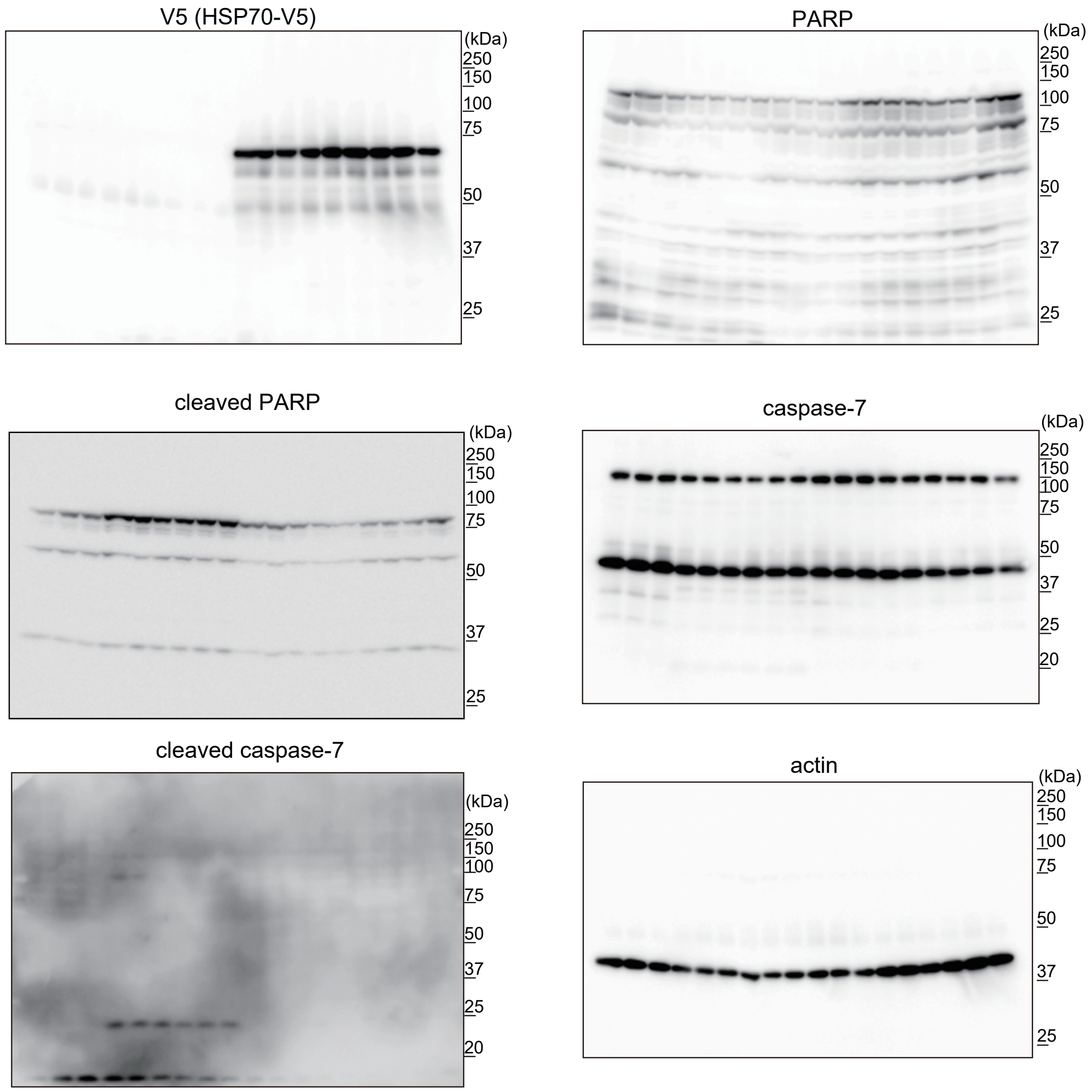

Supplementary Fig. S4.

A

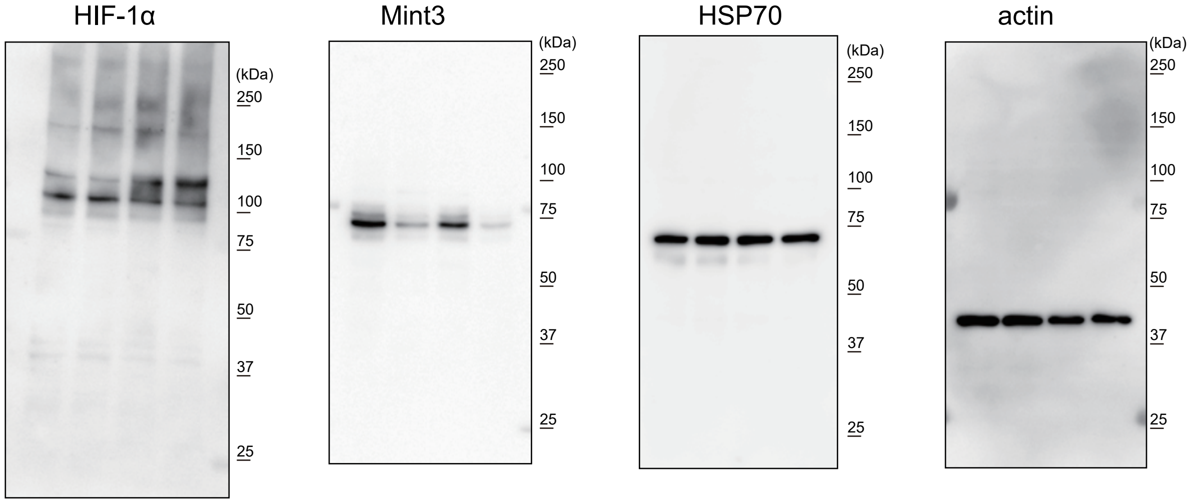

B

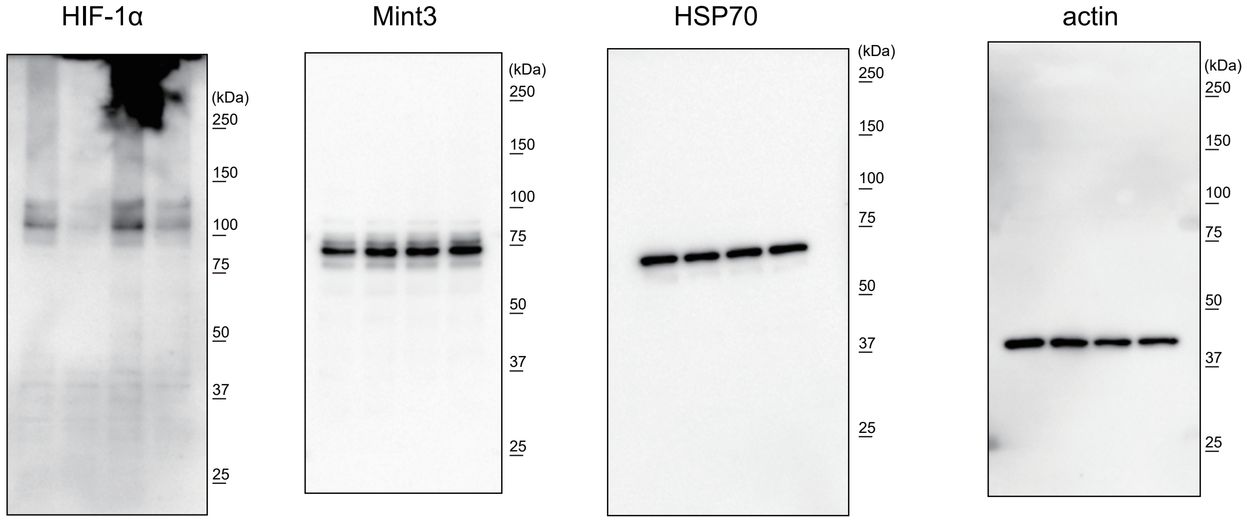

Supplementary Fig. S7

A

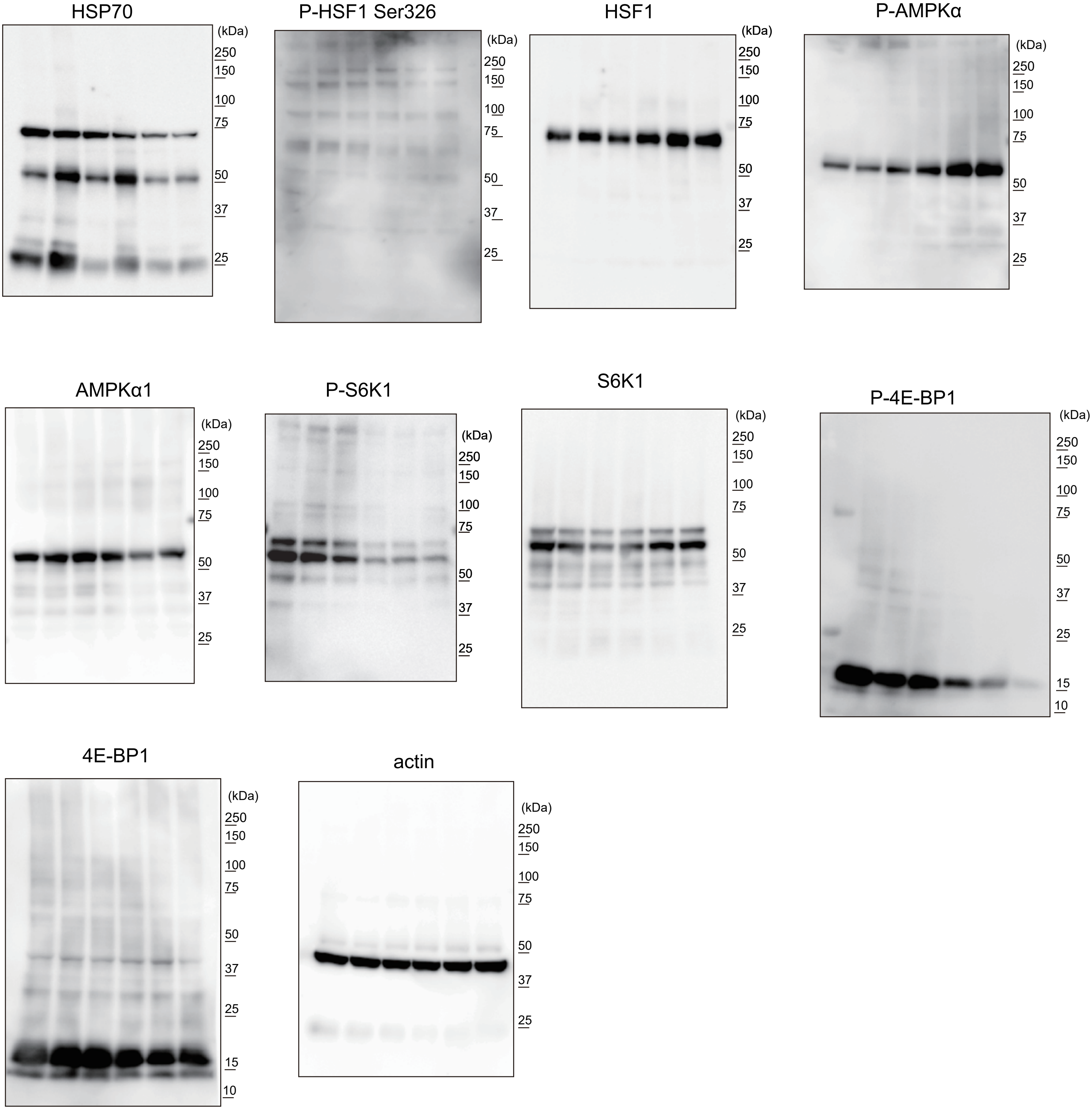

Supplementary Fig. S7

B

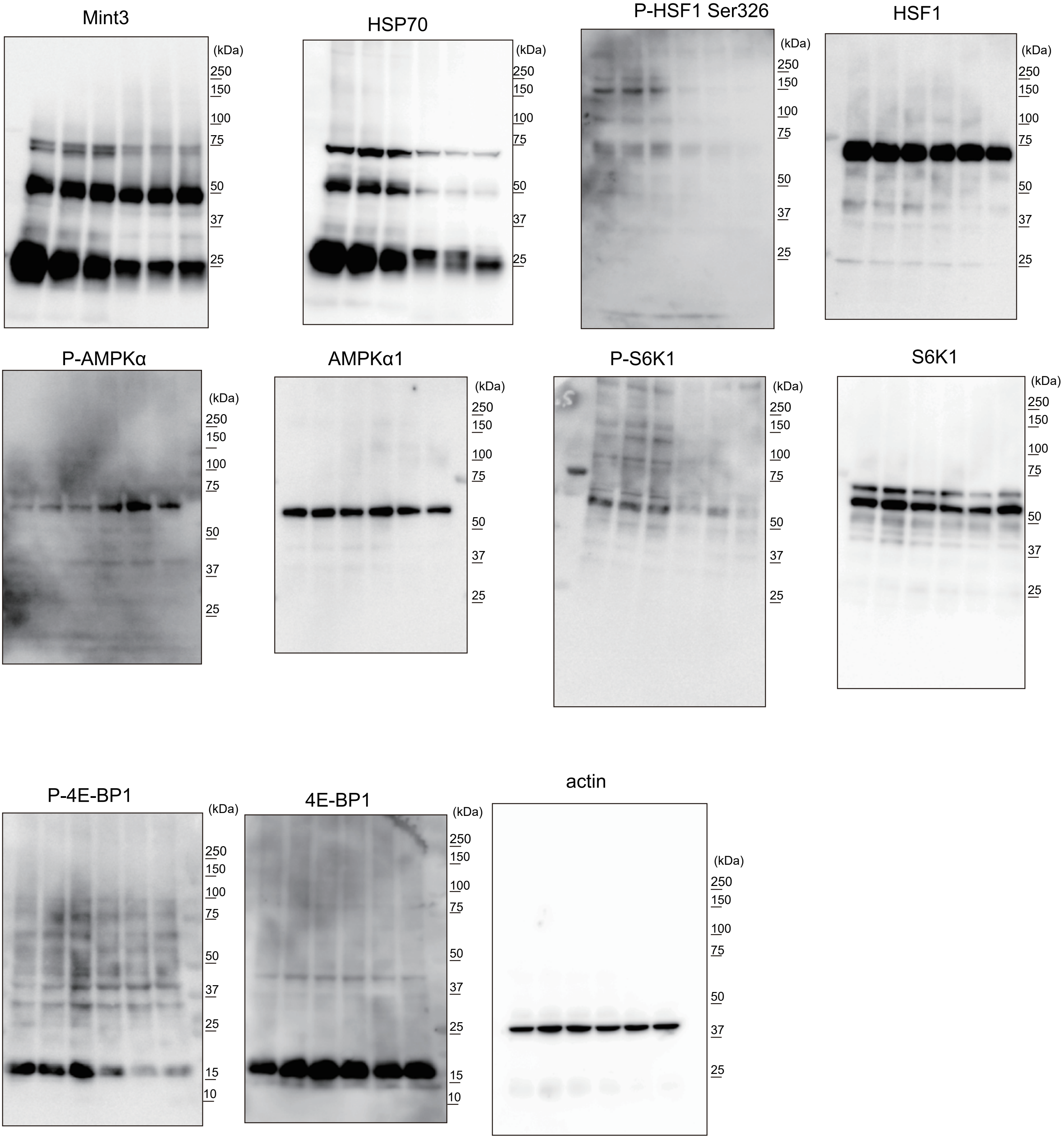

Supplementary Fig. S7

C

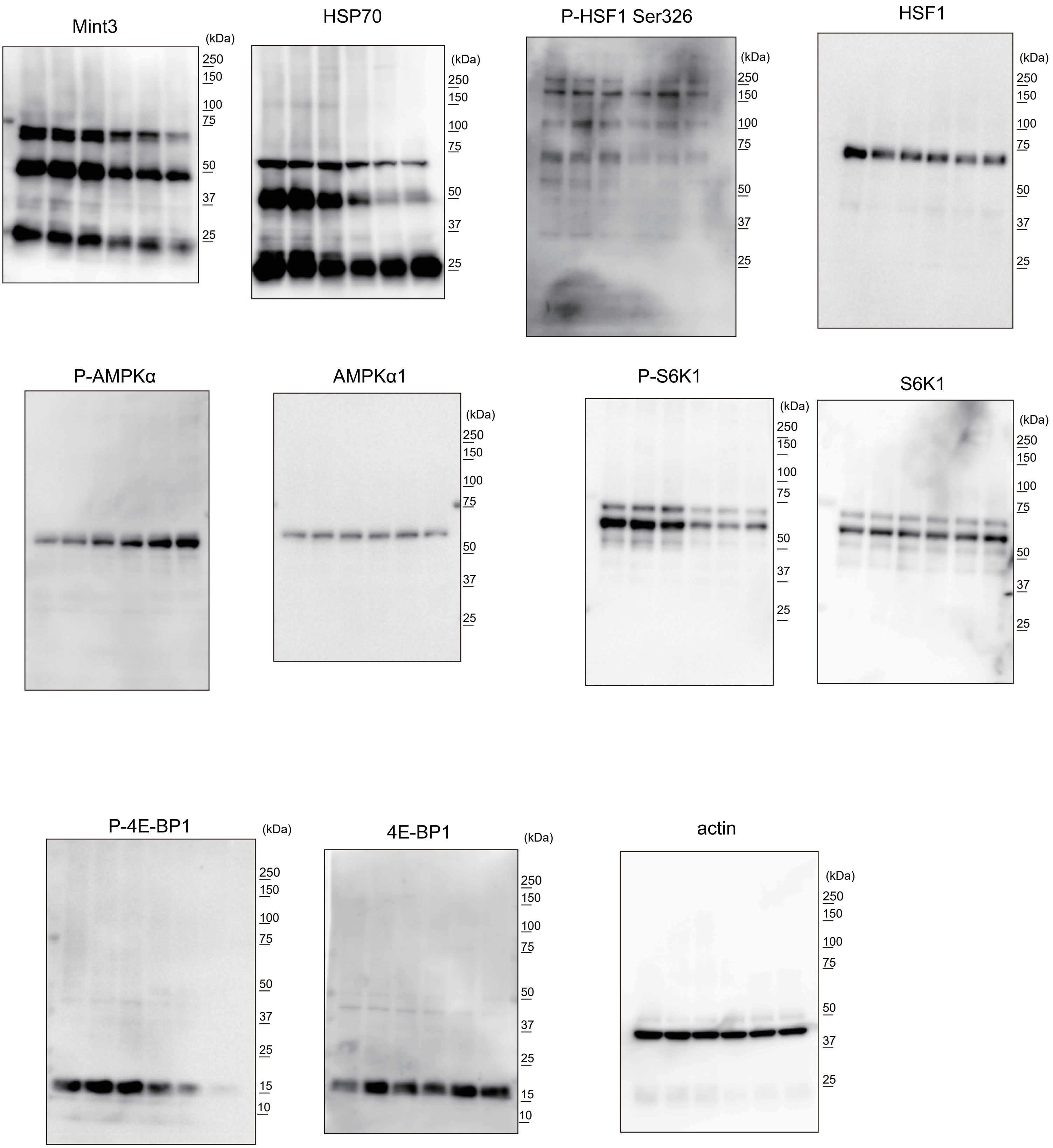

Supplementary Fig. S7

D

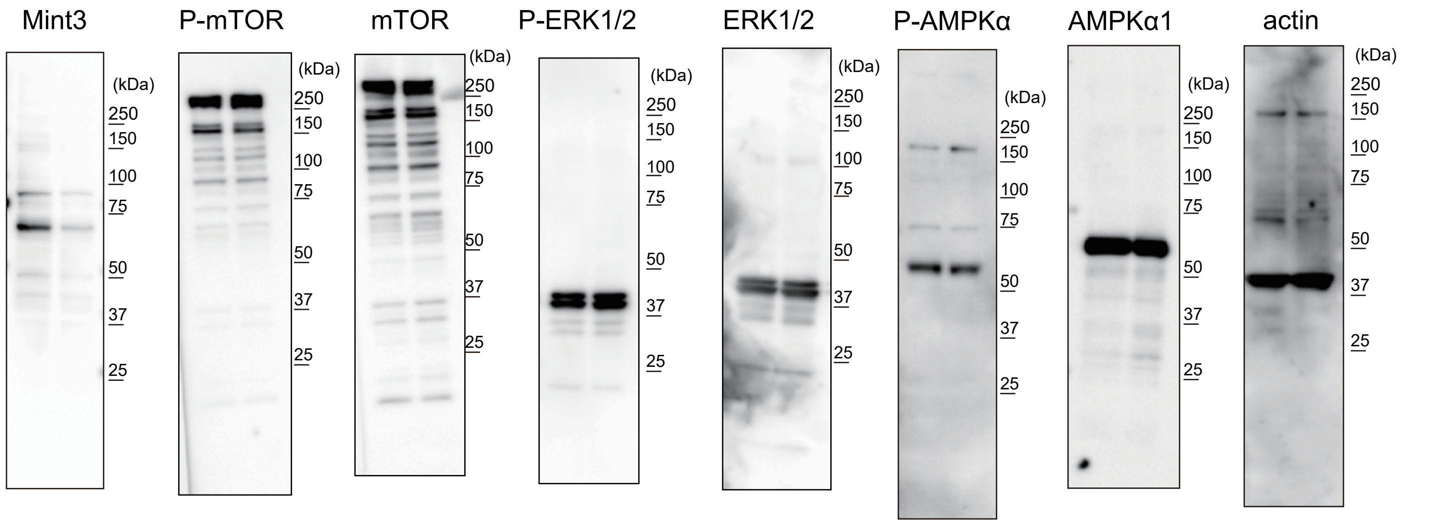

E

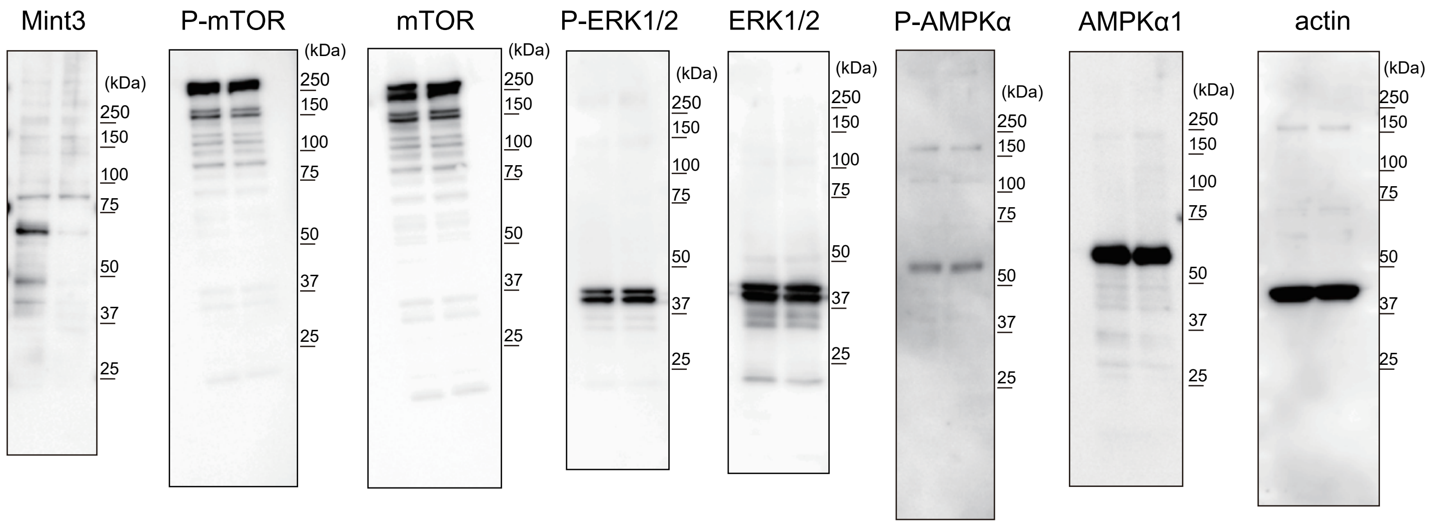

Supplementary Fig. S8

B

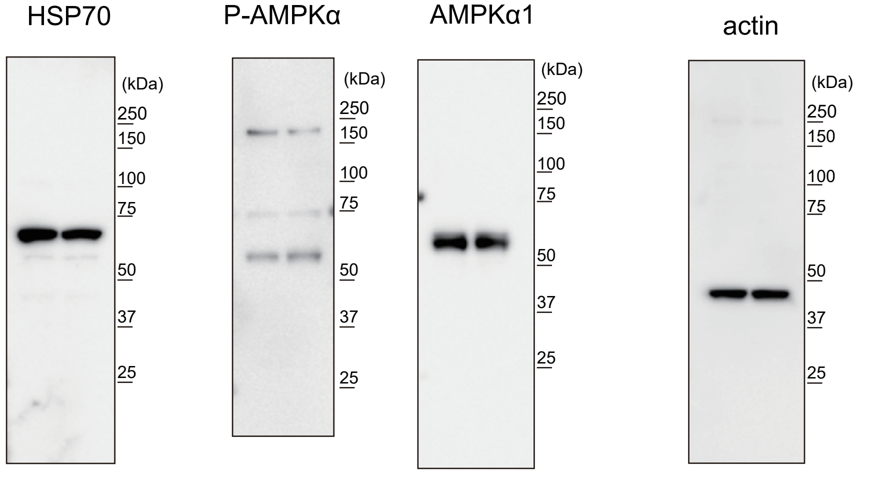

Supplement: Supplementary file 12 — original data [file 41419_2023_6352_MOESM12_ESM.pdf]
